# The ROK kinase *N*-acetylglucosamine kinase uses a sequential random enzyme mechanism with successive conformational changes upon each substrate binding

**DOI:** 10.1016/j.jbc.2023.103033

**Published:** 2023-02-16

**Authors:** Sumita Roy, Mirella Vivoli Vega, Jessica R. Ames, Nicole Britten, Amy Kent, Kim Evans, Michail N. Isupov, Nicholas J. Harmer, GoVV Consortium

**Affiliations:** 1 Living Systems Institute, Exeter, UK; 2 Henry Wellcome Building for Biocatalysis, Biosciences, Exeter, UK

**Keywords:** carbohydrate kinase, enzyme mechanism, magnesium, differential scanning fluorimetry, x-ray crystallography, GlcNAc, *N*-acetylglucosamine, LD, lactate dehydrogenase, MR, molecular replacement, NagK, *N*-acetylglucosamine kinase, GlcNAc-6P, GlcNAc-6-phosphate, NanK, *N*-acetylmannosamine kinase, PK, pyruvate kinase

## Abstract

*N*-acetyl-d-glucosamine (GlcNAc) is a major component of bacterial cell walls. Many organisms recycle GlcNAc from the cell wall or metabolize environmental GlcNAc. The first step in GlcNAc metabolism is phosphorylation to GlcNAc-6-phosphate. In bacteria, the ROK family kinase *N-*acetylglucosamine kinase (NagK) performs this activity. Although ROK kinases have been studied extensively, no ternary complex showing the two substrates has yet been observed. Here, we solved the structure of NagK from the human pathogen *Plesiomonas shigelloides* in complex with GlcNAc and the ATP analog AMP-PNP. Surprisingly, *Ps*NagK showed distinct conformational changes associated with the binding of each substrate. Consistent with this, the enzyme showed a sequential random enzyme mechanism. This indicates that the enzyme acts as a coordinated unit responding to each interaction. Our molecular dynamics modeling of catalytic ion binding confirmed the location of the essential catalytic metal. Additionally, site-directed mutagenesis confirmed the catalytic base and that the metal-coordinating residue is essential. Together, this study provides the most comprehensive insight into the activity of a ROK kinase.

*N*-acetylglucosamine (GlcNAc) is a critical monosaccharide for both prokaryotes and eukaryotes. Eukaryotes widely employ GlcNAc in the *N*- and *O*-linked glycans that decorate protein surfaces; in the glycosaminoglycans hyaluronan, heparin sulfate, and keratan sulfate that form a major part of the connective tissues (1, 2); and in chitin (3). GlcNAc is also used as a reversible modification of proteins (4) that is conserved among metazoans and to decorate some growth factors (5). This modification is particularly common on nuclear proteins and generally acts to modulate signaling (often in competition with phosphorylation) and transcription in response to stress and nutrient conditions (6, 7, 8).

GlcNAc is essential to most prokaryotes, as the cell wall is formed from a polymer of GlcNAc and *N-*acetylmuramic acid cross-linked with peptides (9). Consequently, the key enzymes required for the biosynthesis of the nucleotide-linked sugar UDP-GlcNAc are essential in all bacteria. Many bacteria also require GlcNAc to form their lipopolysaccharides (with GlcNAc forming the core of lipid A) (10) and capsular polysaccharides (11). The first sugar added to the lipid carrier in many oligosaccharides is GlcNAc, its epimer *N-*acetylgalactosamine, or 6-deoxy versions of these (*N-*acetyl-d-quinovosamine and *N-*acetyl-d-fucosamine, respectively) (10, 12, 13). The wzx flippase that transfers oligosaccharides from the cytoplasmic leaflet of the inner membrane into the periplasm (14, 15) and the wzy O-antigen/capsular polysaccharides polymerase (16) have strong specificity for the membrane proximal sugar. Furthermore, most oligosaccharide transferases (17, 18) are exquisitely specific for the *N*-acetyl group, making the *N-*acetylated sugars intimately linked to the surface biology of bacteria.

GlcNAc is generally synthesized by cells from fructose-6-phosphate from central metabolism (Fig. 1) (19, 20). However, many organisms also have pathways for recycling GlcNAc. This is of particular importance for many bacteria that remodel their cell wall and for intracellular bacteria that have a reduced availability of metabolic precursors in their environmental niches. Loss of the recycling pathway enzymes reduces the capacity of bacteria to remodel their cell walls (21, 22, 23, 24). These pathways have been recognized in a wide range of human pathogens (*e.g.*, *Escherichia coli* (22), *Pseudomonas aeruginosa* (25), Enterobacteriaceae, *Staphylococcus aureus* (26, 27), and *Mycobacterium tuberculosis* (28)). Many bacteria utilize chitin as a nutrition resource, using chitinases to recycle it to GlcNAc (29, 30). Chitin is likely to be of particular importance in pathogens of crustaceans and insects (*e.g.*, *Serratia* (31) and *Vibrio* species (32)).

**Figure 1:**
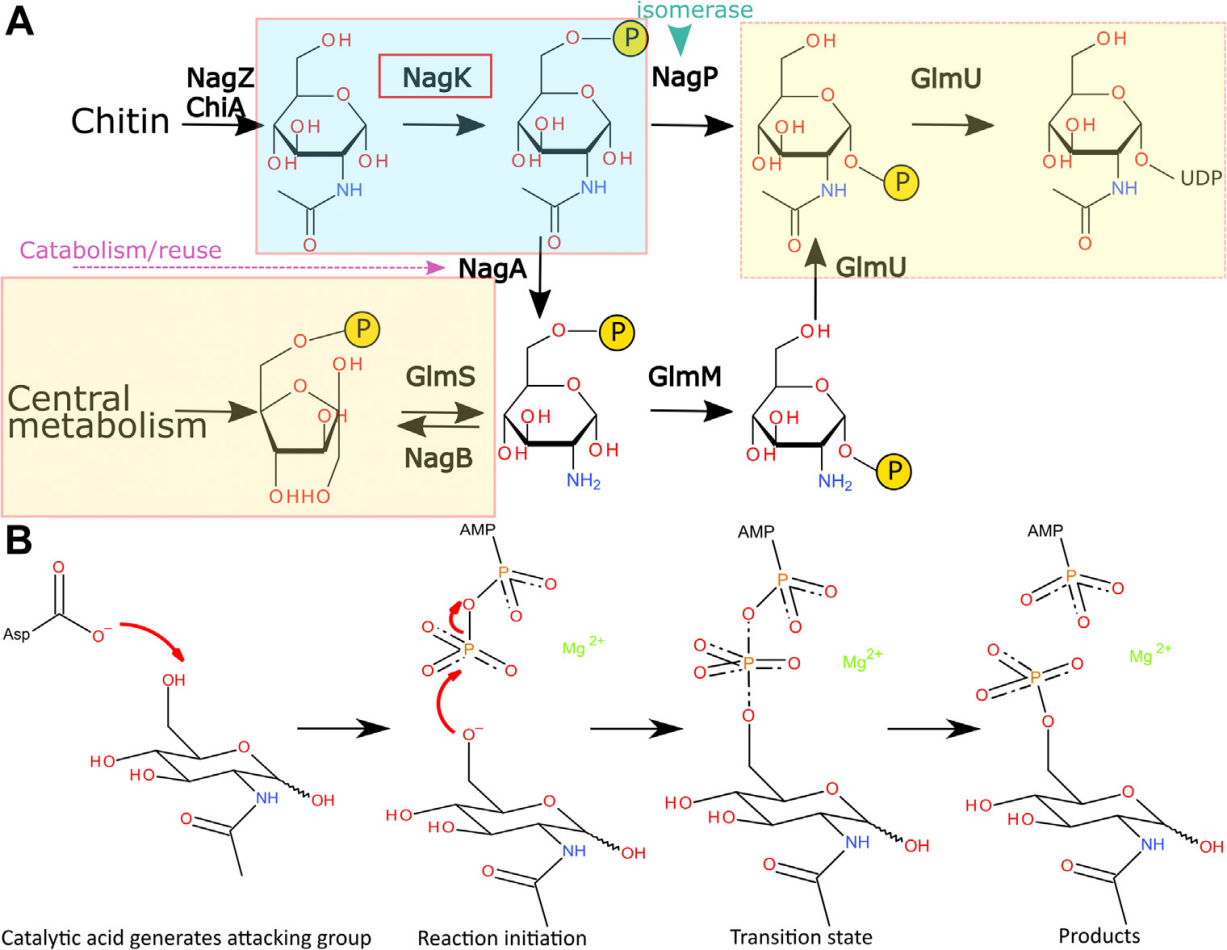
**Biosynthesis and catabolism of *N*-acetylglucosamine.***A*, cells from bacteria to humans synthesize *N-*acetylglucosamine from fructose-6-phosphate derived from the Embden–Meyerhof–Parnas pathway (*orange shaded box*). Some organisms utilize GlcNAc from the environment (*e.g.*, digested chitin, bacterial cell wall components, or glycosaminoglycans). GlcNAc is converted into GlcNAc-6-phosphate by NagK enzymes (*sky blue shaded box*). GlcNAc-6-P can be deacetylated by NagA (*pink dashed arrow*) for catabolism or reuse or isomerized to GlcNAc-1-phosphate by NagP (*green arrowhead*) for direct transfer to UDP (*yellow shaded box*). *B*, mechanism of ROK kinases. ROK kinases are proposed to catalyze phosphate transfer with an aspartic acid residue acting as a general base to deprotonate a hydroxyl. This hydroxyl attacks the ATP γ-phosphate. The transition state (third image) is stabilized by a catalytic cation (here, Mg^2+^). GlcNAc, *N*-acetylglucosamine; NagK, *N-*acetylglucosamine kinase.

An essential step in GlcNAc metabolism is the phosphorylation of GlcNAc to GlcNAc-6-phosphate (GlcNAc-6P). Eukaryotes isomerize this to GlcNAc-1-phosphate (33, 34) (Fig. 1), as their preferred metabolic route to UDP-GlcNAc. In contrast, bacteria that recycle GlcNAc deacetylate GlcNAc-6P, linking recycled and environmental GlcNAc to their central metabolism (35). Phosphorylation of GlcNAc to GlcNAc-6P is performed by a specific kinase, *N-*acetylglucosamine kinase (NagK). Both mammalian (36) and bacterial NagK enzymes belong to the ROK kinase family of carbohydrate kinases (37). This family phosphorylates a broad range of sugars, with individual kinases showing tight specificity for their substrates (38, 39, 40, 41). ROK kinases have a two-domain fold, with the sugar binding between the two domains, causing a structural re-arrangement that forms the active site (42, 43). Other characterized ROK kinases have shown a requirement for either manganese or magnesium for catalysis (40, 44, 45). Existing crystal structures suggest that ROK kinases use a similar mechanism to other classes of carbohydrate kinases (37) (Fig. 1*B*). A conserved aspartic acid side chain deprotonates the 6′-hydroxyl of GlcNAc. This hydroxyl attacks the ATP γ-phosphate, passing through a negatively charged transition state that is stabilized by the catalytic metal. However, current structural information does not include a structure of an ATP analog with an intact γ-phosphate. There is only one structure (from the human *N-*acetylmannosamine kinase [NanK]) that contains a catalytic metal: the metal binding site has not been confirmed by mutations or in bacterial enzymes (36, 46).

Here, we report the activity, structure, and mechanism of NagK from *Plesiomonas shigelloides*. Surprisingly, the enzyme displays a random sequential mechanism, with both GlcNAc and ATP able to bind to the enzyme first. PsNagK showed activity with magnesium and manganese as divalent cofactors. The structure of PsNagK in complex with GlcNAc and the ATP analog AMP-PNP demonstrates how the enzyme catalyzes phosphorylation of GlcNAc. Molecular dynamics simulations allowed us to confirm the location of the catalytic cation binding site. Comparing the ternary complex to the product complex of NagK bound to GlcNAc-6P highlights a possible catalytic mechanism. This provides, for the first time, a comprehensive kinetic and structural characterization of a ROK kinase.

## Results

### NagK activity from divergent species

The enzymatic activity of NagK has previously been described for *E. coli* (47). We determined the activity for a wider range of enzymes to highlight the diversity in activity from different species. We particularly focused on human pathogens with diverse NagK sequences. AlphaFold structures of these orthologs suggest that they have very similar structures (48, 49, 50). Recombinant NagK was readily purified for a range of human pathogens (Figs. S1 and S2). The enzymes showed a range of activities (Table 1), with NagK from *Photobacterium damselae* showing the highest activity.

**Table 1:** Activity of NagK orthologs from diverse bacteria

Species	*k*_*cat*_ (s^−1^)	*K*_*M app GlcNAc*_ (μM)	*K*_*M app ATP*_ (μM)	Identity to *E. coli* NagK (%)	Enzyme, GlcNAc, and ATP concentrations used (nM, mM, mM)
*Vibrio vulnificus*	95 ± 8	210 ± 60	41 ± 9	54.5	6.3, 2, 1
*Photobacterium damselae*	400 ± 20	64 ± 10	250 ± 10	53.8	4.23, 2, 1
*Pseudoalteromonas* sp. *P1-8*	25 ± 1	1100 ± 200	3000 ± 700	40	13.4, 25, 20
*Plesiomonas shigelloides*	202 ± 3	98 ± 6	290 ± 20	59.7	5.1, 2, 1

NagK orthologs from diverse bacteria were purified (Fig. S1) and their activity determined. *k*_*cat*_ and *K*_*M app*_ for both substrates were determined. The table indicates the concentration of NagK used, and the concentration of substrate used for determining *K*_*M app*_ for the reciprocal substrate. The sequences were aligned using MUSCLE and percentage identities calculated in Geneious v. 8.

### NagK uses a sequential mechanism

**Figure 2:**
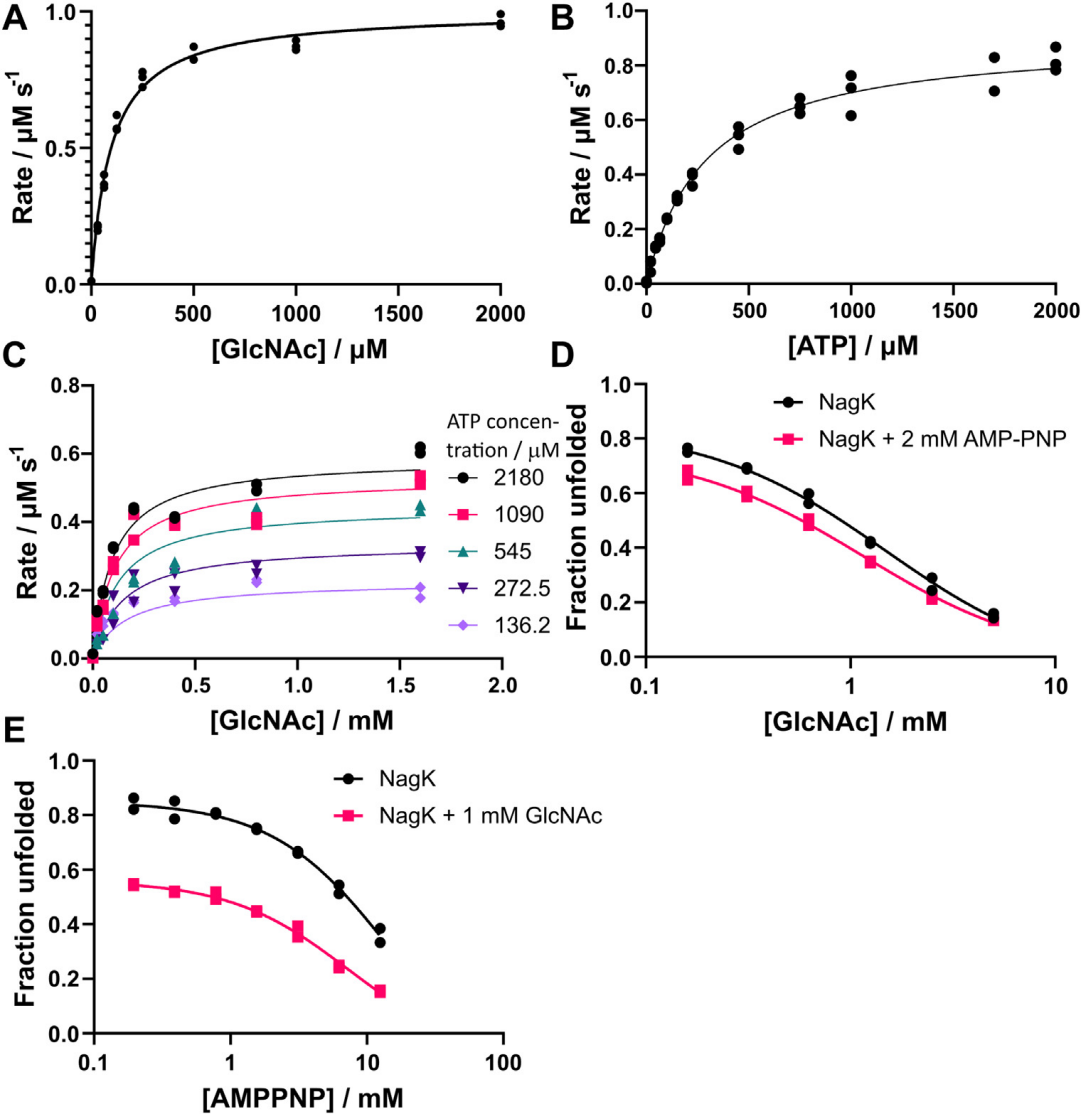
**NagK uses a sequential mechanism of binding.** NagK from *Plesiomonas shigelloides* was selected for a more detailed study of function as it has intermediate activity. *A* and *B*, PsNagK activity fitted to the Michaelis-Menten equation for both GlcNAc (*A*) and ATP (*B*), giving *K*_*M app*_ values of 98 ± 6 μM and 290 ± 20 μM, respectively. Experiments used 5.1 nM PsNagK and 2 mM GlcNAc or 1 mM ATP to determine *K*_*M app*_ for the reciprocal substrate. *C*, testing of both substrates together showed a strong preference to the equation for sequential binding rather than a ping-pong mechanism (Akaike’s information criteria difference = 10.79; *p* = 0.0045). Neither GlcNAc-6-P nor ADP showed product inhibition at readily testable concentrations (Fig. S3), preventing determination of whether the binding is ordered or random. These experiments used 90 ng/ml PsNagK. *D* and *E*, differential scanning fluorimetry of NagK in the presence of GlcNAc and the ATP analog AMP-PNP. The apparent *K*_*D*_ value of NagK for GlcNAc shows no significant difference in the absence (230 ± 20 μM) or presence (270 ± 20 μM) of 2 mM AMP-PNP. The apparent *K*_*D*_ value of NagK for AMP-PNP increases slightly from 2.2 ± 0.6 mM to 3.2 ± 0.5 mM in the presence of 1 mM GlcNAc. All experiments show results representative of at least two experiments performed on different days, with three experimental replicates per datapoint for panels A and B, and two experimental replicates per datapoint for panels C-E. GlcNAc, *N*-acetylglucosamine; NagK, *N-*acetylglucosamine kinase.

We selected NagK from *P. shigelloides* for a more detailed study of the NagK mechanism. The enzyme kinetics showed a sequential mechanism rather than a ping-pong mechanism (Fig. 2, *A*–*C*; *p* = 0.0045). The products GlcNAc-6P and ADP showed weak inhibition, with Morrison *K*_*i*_ values one to two orders of magnitude higher than the cognate substrate *K*_*M*_ (Fig. S3). These could not be used to differentiate between an ordered and random sequential mechanism. Examination of the relationship between *K*′ (apparent *K*_*M*_) and the partner concentration can be diagnostic (51, 52). For our enzymes, these showed no decrease in *K*′ with increasing partner concentration (Fig. S4), consistent with a random sequential mechanism. However, counter examples showing this pattern that nevertheless have an ordered mechanism have been described (53, 54). We therefore examined whether the binding of either substrate affects binding of the other. Past studies of enzyme mechanisms have investigated substrate binding using methods such as differential scanning fluorimetry (55) or fluorescence anisotropy (56). We used differential scanning fluorimetry to determine the dissociation constants of GlcNAc and the nonhydrolyzable ATP analog AMP-PNP. Using the isothermal differential scanning fluorimetry approach (57), we determined that the *K*_*D*_ for GlcNAc in the absence and presence of AMP-PNP were 230 ± 20 μM and 270 ± 20 μM, respectively (Fig. 2*D* and Fig. S5). We chose a temperature of 68 °C to measure at as this gave the optimal signal to determine *K*_*D.*_ The *K*_*D*_ for AMP-PNP in the absence and presence of GlcNAc were 2.2 ± 0.6 mM and 3.2 ± 0.5 mM, respectively (Fig. 2*E*). The difference between these *K*_*D*_ values and the apparent *K*_*M*_ values is likely due to the higher temperature at which these data were collected. There is no significant increase in the affinity for either substrate in the presence of the other. This confirms that NagK likely uses a random sequential mechanism.

### NagK prefers magnesium as the catalytic metal

**Figure 3:**
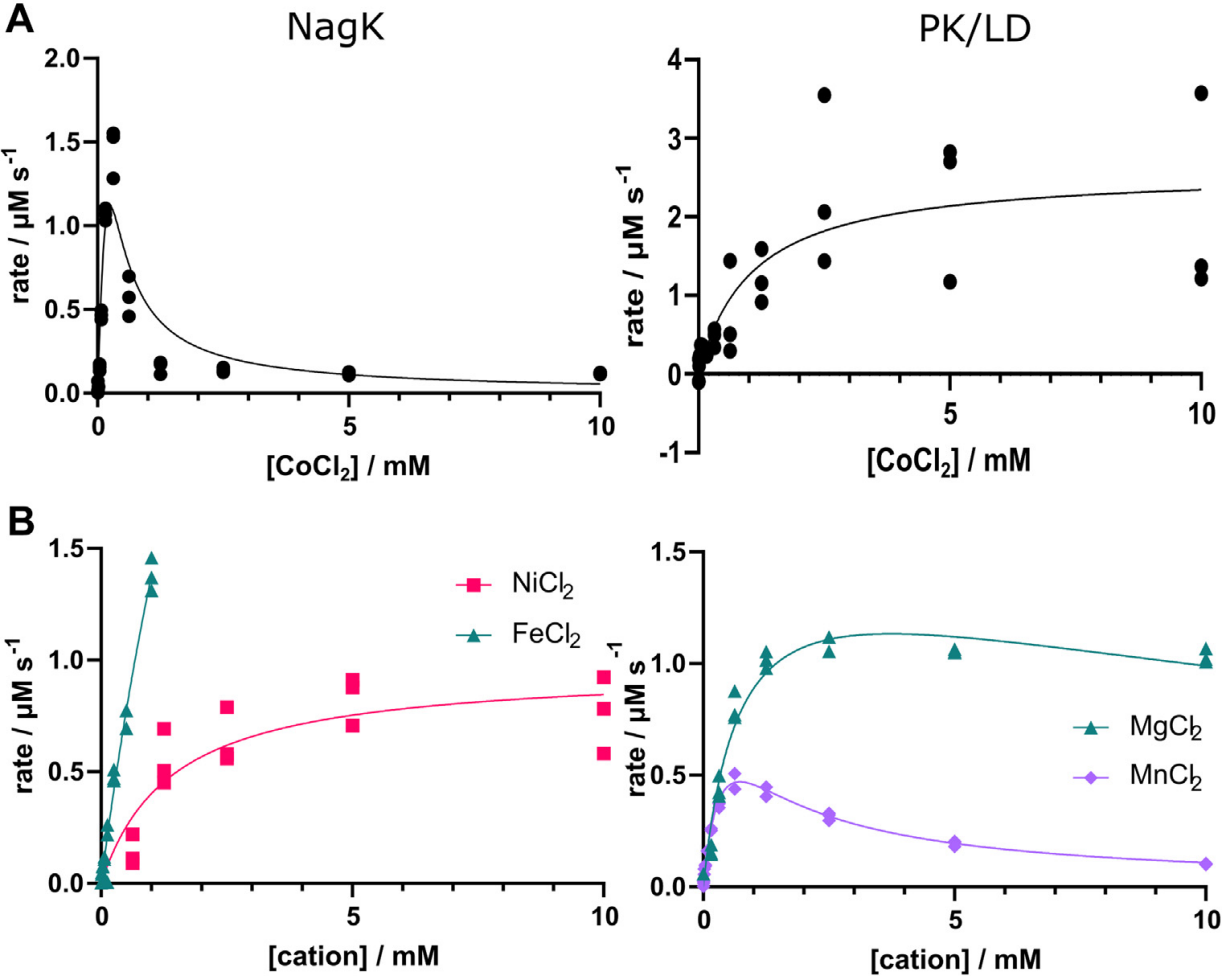
**Magnesium is the preferred metal cofactor of NagK.** The activity of *P. shigelloides* NagK was tested in the presence of diverse cofactors. *A*, cobalt supports the coupling enzymes pyruvate kinase and lactate dehydrogenase at concentrations that give minimal NagK activity. NagK (*left*) shows activity less than 10 times background at concentrations below 20 μM, while supporting much higher rates of the coupling enzyme (*right*) at these concentrations. Conditions used 2 mM GlcNAc, 1 mM ATP, 5.1 nM NagK; for the coupled reaction, 2 U/ml was used; for determination of coupling enzyme efficiency, 0.2 U/ml was used. No activity was observed without divalent cations (Fig. S6). *B*, testing of other cations showed that magnesium, manganese, iron, and nickel all support NagK activity. The saturating concentration for iron is not reached within the constraints of the assay (*left*). Both magnesium and manganese show a substrate inhibition effect (*right*). Nickel shows a maximum rate of 1.0 ± 0.2 μM s^−1^, with a *K*_*½*_ of 1.4 ± 0.4 mM. The maximum rates for magnesium and manganese are 1.1 ± 0.1 μM s^−1^ and 0.47 ± 0.04 μM s^−1^ at 3.7 mM and 0.73 mM, respectively. The inhibition constant *K*_*I*_ is 20 ± 6 mM for magnesium and 1.1 ± 0.1 mM for manganese. Three experimental replicates were taken for each datapoint, and all experiments are representative of at least two experiments performed on different days. GlcNAc, *N*-acetylglucosamine; LD, lactate dehydrogenase; NagK, *N-*acetylglucosamine kinase; PK, pyruvate kinase.

Most carbohydrate kinases require a metal cofactor. The ROK kinases particularly have previously shown a strong requirement for metals. As our coupled enzyme assay is also dependent on metals, we tested the coupling enzymes and the NagK reaction in the presence of the eight divalent cations observed in the M-CSA database (58) (Mg^2+^, Ca^2+^, Mn^2+^, Fe^2+^, Co^2+^, Ni^2+^, Cu^2+^, and Zn^2+^). NagK showed activity with all the cations that support coupling enzyme activity (Fig. S6). However, the NagK rate approaches zero at low Co^2+^ concentrations, where the coupling enzymes retain activity (Fig. 3*A*). This suggests that *Ps*NagK has no activity in the absence of divalent cations. We tested all other relevant divalent cations with 10 μM Co^2+^ to support the coupling enzymes (Table 2 and Fig. 3*B*). No activity was observed with calcium, copper, or zinc. The enzyme showed little preference between manganese (*K*_½_ = 0.47 ± 0.06 mM) and magnesium (*K*_½_ = 0.7 ± 0.1 mM) at low concentrations. Both showed substrate inhibition, with manganese inhibiting at lower concentrations (*K*_i_ = 1.1 ± 0.1 mM; maximum rate 0.47 ± 0.04 μM s^−1^ at 0.73 mM; Fig. 3*B*). Magnesium shows a higher maximum rate (1.1 ± 0.1 μM s^−1^ at 3.7 mM) and would be strongly preferred at physiological concentrations (Fig. 3*B*). Both ferrous iron and nickel also supported NagK activity. Nickel supported activity moderately (maximum 1.0 ± 0.2 μM s^−1^, *K*_½_ = 1.4 ± 0.4 mM). The rate supported by ferrous iron reached 1.4 μM s^−1^ at 1 mM and was clearly not saturating. In both cases, 1 mM represents a far higher concentration than would be found in a bacterial cell, again supporting magnesium as the physiological cation.

**Table 2:** Effect of divalent cations on enzyme activity

Metal	Maximum rate (μM s^−1^)	*K*_*½*_ (mM)	*K*_*I*_ (mM)
Mg^2+^	1.1 ± 0.1	0.7 ± 0.1	20 ± 6
Ca^2+^	0	N/A^a^	N/A^a^
Mn^2+^	0.47 ± 0.04	0.07 ± 0.01	1.1 ± 0.1
Fe^2+^	Not determined	>>1 mM	N/A^a^
Co^2+^	1.2 ± 0.2	>10	<0.05
Ni^2+^	1.0 ± 0.2	1.4 ± 0.4	N/A^b^
Cu^2+^	0	N/A^a^	N/A^a^
Zn^2+^	0	N/A^a^	N/A^a^

All experiments were performed using 180 ng/ml *P. shigelloides* NagK, 2 mM GlcNAc, and 1 mM ATP. Experiments for metals other than cobalt contained 10 μM CoCl_2_ to support coupling enzyme activity. Data for iron and nickel were fitted to the Michaelis–Menten equation. Data for magnesium, manganese, and cobalt were fitted to the substrate inhibition equation. Data were fitted in GraphPad v. 9.4.1.

a This cation did not support NagK activity and so no constants were determined.

b This cation did not show evidence of an inhibitory effect and so no *K*_*I*_ was determined.

### The NagK active site is formed by enzyme closure around the GlcNAc and ATP substrates

**Figure 4:**
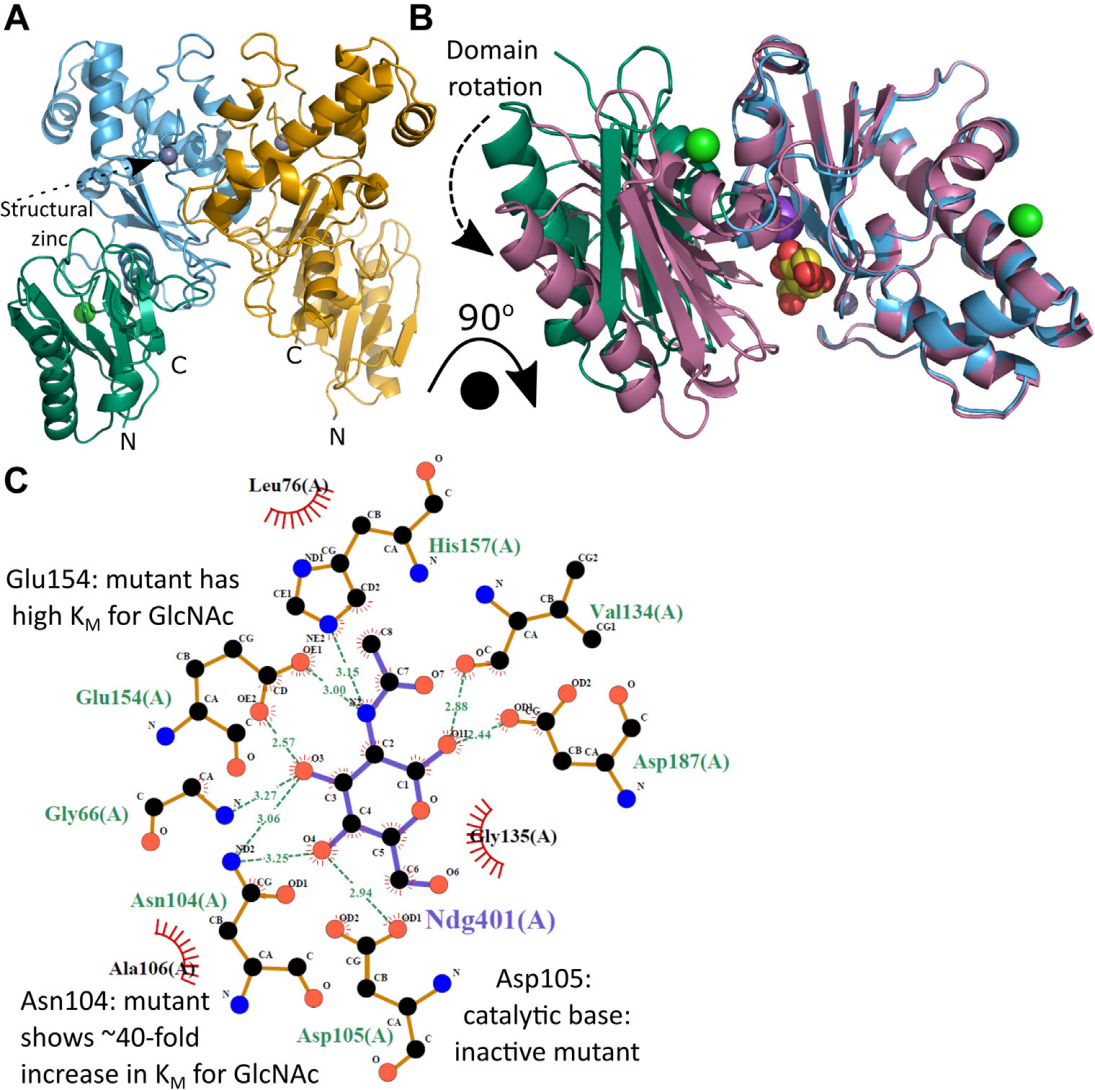
**NagK closes around the *N*-acetylglucosamine substrate.***A*, overall structure of the NagK dimer (PDB: 7P7I). NagK has two domains: an N-terminal small domain (*green*) that includes the C-terminal helix and a large domain (*sky blue*). NagK forms a dimer (second molecule in *yellow*), with the interface between two large domains. A conserved structural zinc ion (*gray sphere*) is seen in the large domain (*dashed arrow*). The N and C termini are indicated. *B*, upon binding of the ligand *N-*acetylglucosamine (*spheres*, carbon atoms in *yellow*), the small domain rotates approximately 15° relative to the large domain to close around the sugar. Unbound structure (7P7I) colored as in A; bound structure (7P9Y) shown in *magenta*. Structures were superimposed over the large domain. *C*, GlcNAc is held in place by a network of amino acids from both small (residues 1–104, 291–303) and large (residues 105–290) domains (7P7Y). Structure images shown as cartoon with ligand atoms shown as *spheres*. Atom colors where not indicated: nitrogen, *blue*; oxygen, *yellow*; chloride, *green*, potassium, *purple*. Panels A and B generated using PyMOL v. 2.4.1 (27); panel C generated using LigPlot+ v2.2 (97, 98). NagK, *N-*acetylglucosamine kinase; GlcNAc, *N*-acetylglucosamine.

Although a structure of *Vibrio vulnificus* NagK has been solved (59), there is no structure of a ligand-bound NagK. We therefore determined the structure of *P. shigelloides* NagK, as this crystallized readily with and without its substrates (Table 3). As expected, *Ps*NagK forms a two-domain fold with a large domain (including the structural zinc characteristic of ROK kinases (37)) and a small domain (Fig. 4*A*). The enzyme closes around the GlcNAc substrate, with the small domain rotating by 23° (moving up to 15 Å) relative to the large domain (Fig. 4*B*). The GlcNAc is bound specifically by the side chains of residues S78, N104, D105, E154, H157, and D187 (Fig. 4*C*).

**Table 3:** Crystal information, data collection, and refinement

Project	NagK	NagK-GlcNAc	NagK-GlcNAc-ADP	NagK-GlcNAc AMP-PNP	NagK-GlcNAc-P	NagK-AMP-PNP
Data collection statistics						
Beamline	I04 Diamond	I03 Diamond	I04–1 Diamond	I04 Diamond	I03 Diamond	I03 Diamond
Wavelength (Å)	0.9795	0.9763	0.9159	0.9795	0.9763	0.9763
Space group	P3_1_21	P3_2_21	P3_2_21	P3_2_21	P3_2_21	P6_5_
Unit cell parameters a, b, c (Å)	95.2, 95.2, 180.6	115.5, 115.5, 119.7.3	115.1, 115.1, 120.3	115.2, 115.2, 120.4	114.5, 114.5, 119.3	121.3, 121.3, 91.7
α, β, γ (°)	90.0, 90.0, 120.0	90.0, 90.0120.0	90.0, 90.0, 120.0	90.0, 90.0, 120.0	90.0, 90.0, 120.0	90.0, 90.0, 120.0
Resolution range (Å)^a^	75.01–1.70 (1.73–1.70)	57.74–1.94 (1.99–1.94)	99.68–1.57 (1.60–1.57)	57.60–2.11 (2.17–2.11)	99.17–1.75 (1.78–1.75)	60.63–2.20 (2.27–2.20)
Total reflections^a^	521,556 (25,890)	457,524 (29,499)	1,273,708 (54,820)	535,990 (43,641)	346,458 (17,705)	127,050 (10,922)
Unique reflections^a^	104,319 (5073)	68,568 (4548)	128,365 (6335)	53,533 (4354)	90,389 (4505)	38,637 (3361)
Completeness (%)^a^	99.6 (99.7)	100.0 (100.0)	100.0 (100.0)	100.0 (100.0)	99.1, (100.0)	99.2 (99.8)
Multiplicity^a^	5.0 (5.1)	6.7 (6.5)	9.9 (8.7)	10.0 (10.0)	3.8 (3.9)	3.3 (3.2)
R_*merge*_ (%)^a^^,^^b^	4.6 (262.5)	8.6 (231.3)	10.1 (321.7)	17.3 (336.0)	9.1 (169.1)	17.8 (272.5)
*<I>/<σ(I)>*^a^	13.8 (0.4)	11.0 (0.8)	12.7 (0.6)	9.5 (0.7)	8.4 (0.8)	5.2 (0.4)
CC_1/2_^a^^,^^c^	0.999 (0.270)	0.999 (0.282)	0.999 (0.291)	0.998 (0.336)	0.998 (0.194)	0.964 (0.396)
Wilson B-factor^d^ (Å^2^)	41.3	48.5	33.4	49.9	35.1	48.9
Refinement statistics						
*R*_*work*_	0.183	0.208	0.185	0.191	0.194	20.9
*R*_*free*_	0.210	0.250	0.212	0.233	0.223	25.2
No. of protein monomers in a.u.	2	2	2	2	2	2
Number of atoms						
Macromolecules	4891	4679	4976	4741	4899	4666
Ligands and metal ions	2	34	86	96	42	64
Solvent	597	382	749	360	511	242
Number of protein residues	609	608	610	610	609	608
RMS bond lengths (Å)	0.009	0.009	0.007	0.007	0.012	0.006
RMS bond angles (°)	1.62	1.67	1.46	1.57	1.77	1.42
Ramachandran favored (%)^e^	97.9	94.4	98.0	96.2	97.9	96.2
Ramachandran outliers (%)^e^	0.0	0.2	0.0	0.0	0.0	0.5
Clashscore^e^	4.05	12.6	8.8	6.9	7.0	6.3
Average B-factor protein (Å^2^)	42.2	55.3	27.8	49.3	35.1	46.5
Average B-factor ligands (Å^2^)	39.2	69.1	30.3	56.8	40.3	72.7
Average B-factor solvent (Å^2^)	55.4	56.9	43.5	57.3	49.6	50.7
RCBS PDB code	7P7I	7P9Y	7P7W	7P9P	7P9L	7PA1

a Values for the highest resolution shell are given in parentheses.

b *R*_*merge*_ = Σ_h_Σ_i_|*I*_*h,i*_ - <*I*_*h*_>|/Σ_h_Σ_i_*I*_*h,I*_.

c CC_1/2_ is defined in (99).

d Wilson B-factor was estimated by SFCHECK (100).

e The Ramachandran statistics and clashscore were calculated using MOLPROBITY (89).

**Figure 5:**
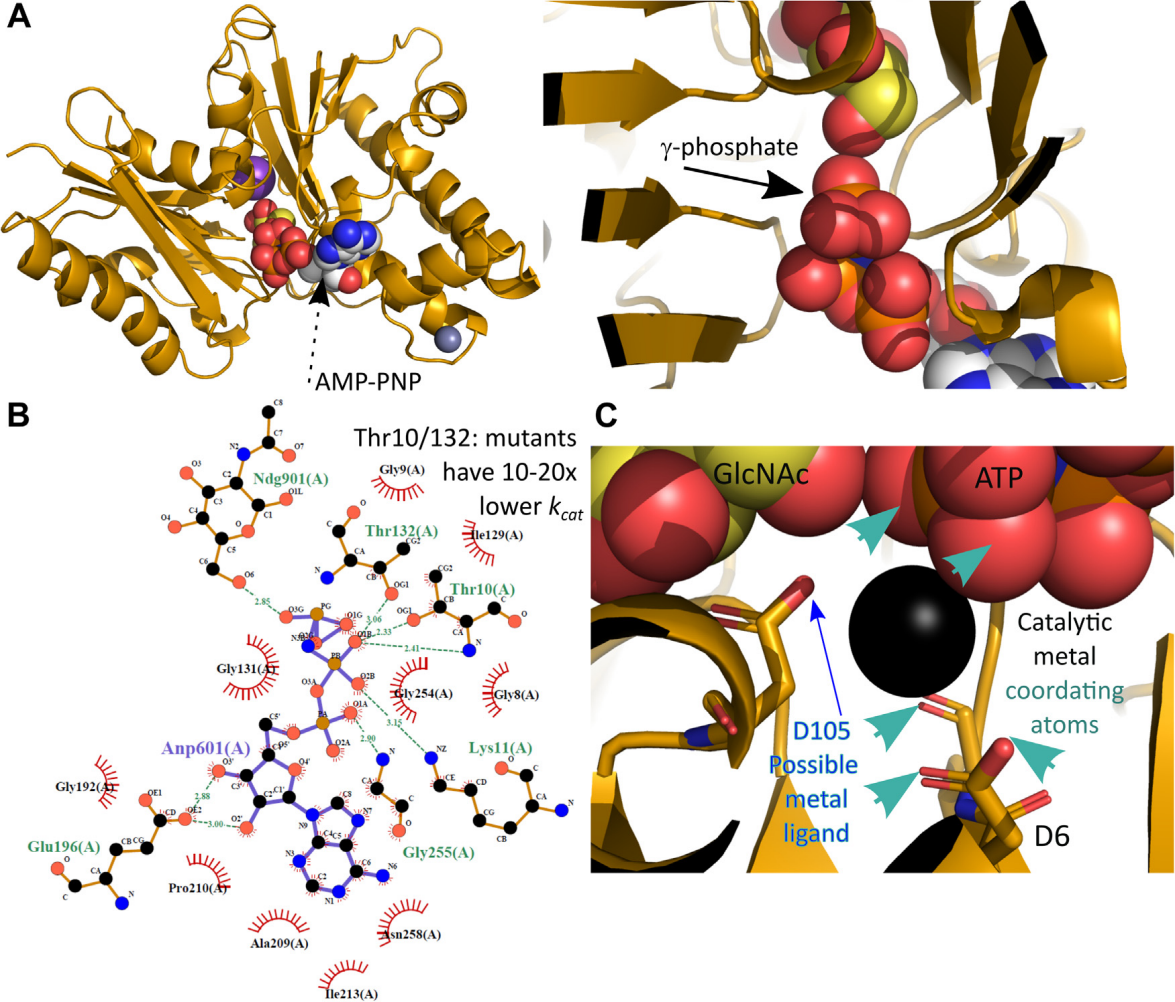
**The NagK ternary complex with GlcNAc and AMP-PNP highlights the likely catalytic mechanism.***A*, structure of the NagK-GlcNAc-AMP-PNP ternary complex (PDB: 7P9P). *Left*: overview of the structure in the same conformation as Fig. 4*B*. The AMP-PNP (*black dashed arrow*) is shown as *spheres* with carbon atoms colored *white*. *Right*: close-up view of the interaction between the two ligands. The terminal phosphate is indicated with the *black arrow*. *B*, AMP-PNP is held in place by hydrophobic contacts to the adenine ring, hydrogen bonds from the ribose ring to E196, and interactions of the phosphate groups with T10, K11, T132, and the protein main chain. *C*, the ternary complex creates a metal binding site that is occupied by water in the structure. Likely water molecule shown as a *black sphere*. Likely metal coordinating atoms (acid oxygens of D6, main chain carbonyl of I7, γ-phosphate atoms) are indicated with *green arrowheads*; the side chain of D105 (*blue arrow*) may act as a sixth ligand. Atom colors where not indicated: nitrogen, *blue*; oxygen, *yellow*; chloride, *green*, potassium, *purple*; zinc, *gray*; phosphorus, *orange*. Panels A and C generated using PyMOL v. 2.4.1 (27); panel B generated using LigPlot+ v2.2 (97, 98). GlcNAc, *N*-acetylglucosamine; NagK, *N-*acetylglucosamine kinase.

We then soaked the ATP analog AMP-PNP into the *Ps*NagK structure. A structure with both GlcNAc and AMP-PNP shows the location of the γ-phosphate in a position poised for catalysis (Fig. 5*A*): the best previous ROK kinase ligand structures showed density only to the β-phosphate (42, 46). The small domain rotates a further 16° to engage the AMP-PNP (Fig. S7*C*). This suggests that the full catalytic complex is formed only when both substrates are bound and that there is a “two-step” closing of the gap between two domains on binding of each substrate. Consistent with this, binding of AMP-PNP alone caused only a small closing of the gap between the domains (Fig. S7*D*). In contrast, structures solved with products showed a conformation similar to the ternary complex. Both the abortive complex of NagK bound to GlcNAc and ADP (Fig. S7*E*), and the complex of NagK with GlcNAc-6P (Fig. S7*F*) resemble the GlcNAc-AMP-PNP complex. AMP-PNP is held in place by the side chains of residues T10, D105, T132 and E196, with the phosphates being coordinated by the main chain of G9, T10, and G255 (Fig. 5*B*). Most of these side chains are well conserved amongst NagKs, consistent with a role in substrate binding (Fig. S8).

We were unable to obtain a structure that contained the catalytic cation. However, our ternary complex with GlcNAc and AMP-PNP is structurally very similar to the previously solved NanK structure that included a catalytic magnesium ((46); Fig. S9). The cation binding site is adjacent to a water molecule in our structure coordinated by D6, the main chain carbonyl of I7, and the γ-phosphate (Fig. 5*C*). To test the hypothesis that this is the metal binding site, we performed molecular dynamics simulations of the active site with divalent cations added in this location and AMP-PNP replaced by ATP. Molecular dynamics of the solved structure over 5 ns showed no significant changes in the structure, aside from a minor re-arrangement of the ATP phosphates (Fig. S10*A*). When magnesium, manganese, or calcium was added to the protein structure, the cation and ATP phosphates re-arrange to form a binding site for the divalent cation. Counterintuitively, in the cases of magnesium and manganese, the re-arrangement brings the cation close to the side chain of D105 and the GlcNAc 6'-O as well as the D6 side chain, I7 main chain carbonyl, and the γ-phosphate (Fig. S10, *B*, *D*). These cations are coordinated to five ligands as one face is partially blocked by the side chain of I127. In contrast, the calcium ion forms a classical octahedral coordination with the side chains of D105 and D6 (both oxygens), I7 main chain, and two oxygens from the ATP γ-phosphate. In this case, GlcNAc 6'-O is excluded from the coordination. This may reduce the acidity of the GlcNAc 6'-O, consistent with calcium not supporting catalysis. The rapid, reproducible re-arrangement of the active site under molecule dynamics strongly supports the hypothesis that this is the cation binding site. However, it is likely that a further re-arrangement of the enzyme active site is necessary for catalysis, as the ATP γ-phosphate remains too far away from GlcNAc 6'-O (4.3 Å) to support a reaction.

### Confirmation of proposed ligand-interacting residues by site-directed mutagenesis

Site-directed mutagenesis of proposed ligand-binding and catalytic residues support the role of these amino acids in *Ps*NagK activity. Mutation to either D105N (catalytic base) or D6N/A (metal coordinating negatively charged group) results in a loss of activity below the limit of detection (at least 1000-fold; Table 4). Mutation of the phosphate coordinating T10V and T132V results in a loss of activity, without substantially affecting the *K*_*M*_ for either substrate. Mutation of some side chains that coordinate GlcNAc (N104D, E154Q or double mutant, and D187N) results in substantial increases in *K*_*M*_ for both substrates; for the E154Q mutants, the rate is substantially reduced. Mutation of other conserved GlcNAc binding residues S78A and E196Q resulted in clear increases in rate without affecting *K*_*M*_. These two residues are not well conserved (Fig. S8), and the residues mutated to are found in other orthologs. We did not mutate H157 as this residue also coordinates to the structural zinc atom, and mutation would likely significantly affect the protein structure; all the tested mutants showed good stability in differential scanning fluorimetry (Fig. S12).

**Table 4:** Effect of mutants of ATP and GlcNAc binding residues

*Ps*NagK variant	*k*_*cat*_/s^−1^	*K*_*M app GlcNAc*_ (μM)	*K*_*M app ATP*_ (μM)	*k*_*cat*_/*K*_*M app GlcNAc*_ (μM^−1^ s^−1^)	*k*_*cat*_/*K*_*M app ATP*_ (μM^−1^ s^−1^)	Enzyme, GlcNAc, and ATP concentrations used (nM, mM, mM)
Wildtype	202 ± 3	98 ± 6	290 ± 20	2.1 ± 0.1	0.70 ± 0.05	5.1, 2, 1
D6A	N/A^a^					Attempted up to 82 nM with 2 mM GlcNAc, 1 mM ATP
D6N	N/A^a^					As for D6A
T10V	12.5 ± 0.2	157 ± 10	420 ± 50	0.080 ± 0.005	0.030 ± 0.004	82, 0.8, 1
S78A	416 ± 9	126 ± 7	130 ± 10	3.3 ± 0.2	3.2 ± 0.3	2.5, 0.8, 1
N104D	364 ± 36	4400 ± 300	3000 ± 700	0.08 ± 0.01	0.12 ± 0.03	28, 30, 5
D105N	N/A^a^					As for D6A
T132V	18.4 ± 0.3	200 ± 20	220 ± 10	0.092 ± 0.009	0.084 ± 0.004	170, 2, 1.1
E154Q	12.3 ± 1.5^b^	*K*_*M*_*increases*	2400 ± 600	N/A^b^	0.005 ± 0.001	82, 30, 2; 4 U/ml PK/LD used
D187N	110 ± 2.2	430 ± 20	580 ± 90	0.26 ± 0.01	0.19 ± 0.03	14, 2, 5
E196Q	665 ± 15	230 ± 20	200 ± 20	2.9 ± 0.3	3.3 ± 0.3	5.1, 1.2, 1
N104D E154Q	6.4 ± 0.9^b^	*K*_*M*_*increases*	1200 ± 500	N/A^b^	0.005 ± 0.002	280, 30, 10

Site-directed mutants were prepared for *P. shigelloides* NagK at key side chains that coordinate ATP, GlcNAc, or magnesium. *k*_*cat*_ and *K*_*M app*_ for both substrates were determined as for the wildtype enzyme. The table indicates the concentration of NagK used, and the concentration of substrate used for determining *K*_*M app*_ for the reciprocal substrate.

a D6A, D6N and D105N mutants caused a loss of activity below the limit of detection of the assay (*k*_*cat*_ < 0.01 s^−1^); no kinetic constants could be determined.

b The E154Q and N104D/E154Q mutants caused the apparent *K*_*M*_ for GlcNAc to increase to above 50 mM (*i.e.*, the plot of rate against substrate concentration was a straight line). An apparent *k*_*cat*_ at 50 mM GlcNAc was determined; *K*_*M app GlcNAc*_ and *k*_*cat*_/*K*_*M app GlcNAc*_ cannot be determined.

## Discussion

GlcNAc recycling from the cell wall is important for the biology of many human pathogens. These include some of the ESKAPE pathogens (60) of greatest concern for antimicrobial resistance (22, 25, 26, 27). To efficiently recycle cell wall GlcNAc, bacteria phosphorylate and then de-acetylate GlcNAc to form glucosamine-6-phosphate (35), an intermediate in the essential UDP-GlcNAc biosynthesis pathway (Fig. 1 and (19, 20)). Here, we have thoroughly characterized the first enzyme that performs the first of these steps, NagK. This enzyme belongs to the ROK kinase family of carbohydrate kinases (37). Key questions arising from previous studies of ROK kinases were the order of binding of substrates, confirming the location of the catalytic metal ion and the location of the γ-phosphate.

In common with previous ROK kinases, we determined that NagK has an absolute requirement for divalent cations (40, 45, 61). Magnesium, manganese, iron, cobalt, and nickel support NagK function, while calcium, copper, and zinc do not. Physiologically, magnesium would likely be preferred as bacterial intracellular free magnesium concentrations (∼2 mM) exceed *K*_½_ (0.7 mM), while free iron and manganese concentrations (1–15 μM) are well below concentrations where these support a high NagK rate, while only low micromolar concentrations of nickel and cobalt are tolerated by bacteria (62, 63, 64, 65). Comparison of the crystal structure of NagK bound to GlcNAc and AMP-PNP to the human NanK structure (46) suggested that the metal ion should bind into a pocket adjacent to the γ-phosphate. This pocket would be coordinated by two oxygens from the γ-phosphate, the main chain carbonyl of I7, and the side chain of D6. An alignment of ROK kinases shows that D6 is strongly conserved as an acidic residue (Fig. S11). This has previously been proposed (albeit with limited evidence) as a metal ion binding residue (36). To support this proposal, we added a magnesium ion to this site in our structure and performed a molecular dynamics simulation. The maintenance of the ion in this location is strongly supported in the simulation, with both magnesium and manganese predicted to coordinate to both substrates. Furthermore, mutation of D6 to either asparagine or alanine completely abolishes the activity of the enzyme. Given that D6 is not close to either substrate in the crystal structure, this very strong phenotype strongly supports a role in binding to the catalytic metal ion. These observations strongly support this pocket as the metal binding site for a wide range of ROK kinases.

The effect of mutations in GlcNAc binding residues is in accordance with previous studies. A detailed phylogenetic study proposed that the 3′-OH is coordinated by asparagine (N104) and glutamic acid (E154) residues (39). Mutations in either of these residues significantly reduced the activity of NagK. In contrast, two side chains that contact GlcNAc in the crystal structures (S87 and E196) are not evolutionarily conserved (Fig. S8). Mutation of these side chains increases the catalytic efficiency of NagK *in vitro*. The 1′-OH is engaged by an aspartic acid (D187), mutation of which reduces catalytic efficiency: this is not conserved in *Pseudoalteromonas* NagK and may explain the reduced activity of this ortholog. The highly conserved aspartic acid (here D105) coordinates the 6′-OH, in common with previous NagK structures (39, 41, 42, 43, 46). As was previously observed for human *N*-mannosamine kinase (46), mutation of the aspartic acid coordinating the 6′-OH (here D105) abolishes activity. This strongly suggests that this is the catalytic base (42, 46).

Our structures provide for the first time a complex of a ROK kinase poised for activity. The structure shows the AMP-PNP γ-phosphate positioned above the 6′-OH group of GlcNAc. The catalytic base, D105, is in position to de-protonate the 6′-O and turn this into a strong nucleophile. The location of the phosphate group allows coordination of two oxygens with the catalytic metal ion. Other carbohydrate kinases generally follow a mechanism of a nucleophilic substitution with a negatively charged intermediate stabilized by a metal ion (37, 66, 67, 68). Based on ours and others’ structures, it seems highly likely that ROK kinases follow a similar mechanism.

In conclusion, our study provides further detail explaining the catalytic power of ROK kinases. Our structures demonstrate the choreography of the two enzyme domains as they bind partners to form the ternary complex and release these partners and the location of the critical γ-phosphate in the ternary complex. We demonstrate that a metal ion is required for NagK enzymes and that the conserved ROK kinase metal coordinating acid is essential for enzyme activity. Our data confirm the critical side chains that support NagK binding to its substrate GlcNAc. The availability of a detailed structure of the catalytic state of ROK kinases will enable the engineering of these enzymes to phosphorylate alternative substrates to support synthetic biology. This enzyme would also be an attractive target for the development of small molecule inhibitors to target bacteria that rely on cell wall remodeling as part of their pathogenic processes.

## Experimental procedures

### Construction of expression vectors

A codon-optimized *nagK* gene from *P. shigelloides* was cloned into the pOPINS3C expression plasmid (N-terminal polyhistidine and SUMO tags; Addgene #41115 (69)). The gene sequence was obtained as a gBlock (IDT). DNA was amplified by PCR using the following primers: forward: 5′- cagcggtctggaagttctgtttcagggtacc-3′; reverse: 5′- aagctttctagaccagtttgtgattaacctc-3′. Each PCR reaction contained 1 ng/μl of gBlock DNA, 0.25 μM each primer, 2.5 mM dNTPs, 1× Phusion buffer and 2 U Phusion Polymerase (NEB). The PCR protocol used was an initial denaturation of 30 s at 98 °C, followed by 35 cycles of 10 s at 98 °C, 10 s at 55 °C and 1 min at 72 °C, with a final elongation step of 5 min at 72 °C. The PCR fragment and the plasmid (pOPINS3C) were assembled using the NEBuilder HiFi DNA Assembly kit (NEB), following the manufacturer’s recommendations. The assembled product was transformed into 5-alpha competent cells (NEB), and the insert sequence was confirmed by Sanger sequencing (Source Bioscience). Codon optimized *nagK* genes from *P. shigelloides, V. vulnificus*, *Pseudoalteromonas* sp. *P1-8,* and *P. damselae* were cloned into pNIC28-Bsa4 (N-terminal polyhistidine tag; Addgene #26103 (70)), pOPINS3C, and pGAT2 (N-terminal polyhistidine and GST tags; Addgene #112588; last two genes (71)) by Twist Bioscience. Genbank files for all plasmids are available in Supplementary Data. Plasmids were transformed into the expression strain *E. coli* BL21 (DE3) (Novagen) using ampicillin (100 μg/ml; pGAT2 and pOPINS3C clones) or kanamycin (50 μg/ml; pNIC28 clones) for selection.

### Expression and purification of NagK

NagK was expressed in 1 L of high salt LB broth supplemented with 100 μg/ml ampicillin or 50 μg/ml kanamycin as appropriate. Each flask was inoculated with 10 ml of an overnight culture and grown at 37 °C with shaking at 200 rpm until *A*_600_ reached 0.6. NagK expression was induced with 200 μM isopropyl thio-β-D-galactoside, and cultures were grown at 20 °C for 18 h. Cells were harvested by centrifugation at 4500 × *g* for 30 min at 4 °C. The pellet was resuspended in binding buffer (20 mM Tris-HCl, 500 mM NaCl, and 10 mM imidazole, pH 8.0) and lysed by sonication (SONIC Vibra cell VCX130). The lysed sample was clarified by centrifugation (24 000 × *g* for 30 min at 4 °C). The soluble fraction was purified using an ÄKTAxpress chromatography system (GE Healthcare). The sample was purified firstly using a 1 ml HisTrap crude column (GE Scientific). After loading sample, the column was washed with binding buffer, and the protein eluted into binding buffer with imidazole at 250 mM. The product was purified over a Superdex 200 16/60 size-exclusion column (GE Healthcare) and eluted isocratically into 10 mM Hepes, 500 mM NaCl, pH 7.5. The eluted protein was concentrated using a Vivaspin centrifugal concentrator (Generon) to 1 mg/ml and stored at −20 °C with 20% (v/v) glycerol for enzymatic assays or concentrated to 11.5 mg/ml and stored at −80 °C in small aliquots without any glycerol for crystallization. Protein concentration was determined using a Nanodrop 2000 nanospectrophotometer (Thermo). The extinction coefficient for NagK was determined using the Protparam tool (https://web.expasy.org/protparam/) (72).

### Kinetic analysis

NagK activity was assayed using the previously described coupling reaction with pyruvate kinase (PK) and lactate dehydrogenase (LD (73)). For *P. shigelloides*, the His-tagged protein was used. Reactions contained 90 to 6000 ng/ml NagK, 40 mM Hepes, pH 7.5, 100 mM KCl, 8 mM MgCl_2_, 5 mM DTT, 100 μg/ml BSA, 200 μM NADH, 500 μM phosphoenolpyruvate, 2 U/ml PK-LD (Merck #P0294), 2 mM GlcNAc, and 1 mM ATP. Reactions were performed in 96 well flat-bottomed plates (Greiner #655001) in a total reaction volume of 200 μl. Reactions were monitored by measurement of the absorbance at 340 nm over 40 min in an Infinite M200PRO plate reader (Tecan) with incubation at 37 °C. Datasets were examined individually to determine the region of each experiment that corresponds to the initial rate. Three experimental replicates were performed for all reactions.

Kinetic parameters (*K*_M_ and *k*_cat_) for ATP and GlcNAc were determined by varying either ATP or GlcNAc concentrations between 2 to 0.02 mM and 2 to 0.03 mM, respectively, at constant concentrations of enzyme and partner substrate (values detailed in figure/table legends). The data were fitted to the Michaelis–Menten equation in Prism 9.0.1 (GraphPad). To determine the substrate mechanism, the initial reaction rates were measured with a two-fold dilution of GlcNAc from 2 mM in eight steps and with a two-fold dilution of ATP from 2180 μM in five steps. Two experimental replicates were taken for each data point. Data were fitted to the sequential bi-bi and ping-pong Equations (1), (2) in Prism 9.0.1 (GraphPad) (73, 74, 75). To determine the effect of divalent cations, the coupling enzymes were first tested in a mixture of 40 mM Hepes, pH 7.5, 100 mM KCl, 5 mM DTT, 100 μg/ml BSA, 200 μM NADH, 500 μM phosphoenolpyruvate, 1 mM ADP, and 0.2 U/ml PK-LD. MgCl_2_, CaCl_2_, MnCl_2_, FeCl_2_, CoCl_2_, NiCl_2_, CuCl_2_, and ZnCl_2_ were tested at 1 to 8 mM. 180 ng/ml NagK was initially tested in the same conditions with ADP substituted with 2 mM GlcNAc, 1 mM ATP, and 10 μl CoCl_2_, and the PK-LD increased to 1 U/ml. *K*_M_ and *V*_max_ were determined for MgCl_2_, MnCl_2_, FeCl_2_, CoCl_2_, and NiCl_2_ by varying the concentration between 0 and 10 mM (0–1 mM for FeCl_2_), in the same conditions. Three experimental replicates were performed for all reactions. The data were fitted to the Michaelis–Menten equation or substrate inhibition Equation (3) as appropriate in Prism v. 9.0.1.

(1) $$v=\frac{V_{\max}\left[A\right]\left[B\right]}{K_{iA}K_{B}{+K}_{A}\left[B\right]{+K}_{B}\left[A\right]+\left[A\right]\left[B\right]}$$

(2) $$v=\frac{V_{\max}\left[A\right]\left[B\right]}{K_{A}\left[B\right]{+K}_{B}\left[A\right]+\left[A\right]\left[B\right]}$$

(3) $$v=\frac{v_{\max}\left[S\right]}{K_{M}+\left[S\right]\left(1+\frac{\left[S\right]}{K_{1}}\right)}$$

### Differential scanning fluorimetry

The dissociation constant (*K*_*D*_) for NagK with its substrates was determined using differential scanning fluorimetry (57). Each sample contained 0.1 mg/ml NagK, 8× SYPRO Orange dye (Fisher Scientific #10338542), 10 mM Hepes pH 7.5, 100 mM KCl, and varying concentrations of either GlcNAc, AMP-PNP, or the combination of these in a total volume of 10 μl. Data were collected on a Rotorgene Q (Qiagen) using the ROX channel to collect data. The melt curves showed a monotonic melt. Raw data were converted to a percentage unfolded using the fluorescence readings at the start and end of the melt to define 0 and 100% unfolded. 68 °C was selected as the temperature giving an optimal range of unfolding percentages. Data were fitted to Equation 4 using GraphPad v. 9.0.1.

(4) $$f_{u}=Bottom+\left(Top-Bottom\right)\left(1+\frac{\left[S\right]}{{EC}_{50}}\right)$$

Where *f*_*u*_ is the fraction unfolded, Top and Bottom are the maximum and minimum unfolded fractions, [S] is the varied substrate concentration, and EC_50_ is the substrate concentration that reduces the unfolded fraction by half. Equations fixing Bottom as zero and including a Hill slope were rejected as inferior to three for these data based on Akaike’s information criterion.

The fitted EC_50_ values were converted to *K*_*D*_ using Equation 5 (57).

(5) $$K_{D}=\left({1-f}_{u0}\right)\left({EC}_{50}-\frac{{\left[p\right]}_{T}}{2}\right)$$

where *f*_*u0*_ is the fraction unfolded at zero substrate concentration, and [P]_T_ is the total protein concentration.

For verifying mutant stability, the melting temperature was determined using the Boltzmann method using Protein Thermal Shift Software v. 1.4 (Applied Biosystems).

## Crystallization

For crystallization, the His-SUMO tagged *P. shigelloides* NagK was used. Crystals were grown using the microbatch method using an Oryx8 crystallization robot (Douglas Instruments). Initial crystals grew in well E6 of the Morpheus I screen (Molecular Dimensions), mixed 1:1 with 5 mg/ml NagK. Seed stocks were prepared from these crystals in 0.1 M Mops pH 7.5, 30% (v/v) ethylene glycol, and 10% (w/v) PEG 8000. Final crystals were grown by matrix microseeding (76) these crystals into the Morpheus I screen using a mix of 3:2:1 5 mg/ml NagK: mother liquor: seeds. The successful crystallization conditions, soaking conditions, and cryoprotectants used are detailed in Table S1.

### X-ray data collection and structure determination

Data were collected at Diamond Light Source (Didcot) at 100 K using Pilatus 6M-F detectors and wavelengths of 0.92 to 0.98 Å. All data were processed using XDS (77). Further data processing and structural studies was carried out using CCP4 program package (78, 79). The apo structure of NagK was solved by the molecular replacement (MR) using the MR pipeline MORDA (80) with the best solution found for the model (PDB ID: 4DB3). The model was refined using REFMAC5 (81) and *PHENIX* (82) and rebuilt using COOT (83). The refined apo NagK model was used as a MR search model in MOLREP (84) for the NagK-GlcNAc-ADP data, which crystallized in a different space group. The MR solution was refined using *Buccaneer* (85), following which further refinement was performed as above. The crystals of NagK-GlcNAc-AMP, NagK-GlcNAc, NagK-GlcNAc-6′-phosphate, and NagK-GlcNAc-AMP-PNP were in the same space group as the NagK-GlcNAc-ADP complex; however, phased MR (86) was used to reposition the small domain in the NagK-GlcNAc structure. All structures were subjected to phased refinement in REFMAC5 (87) with input density modification phases (88) from non-crystallographic symmetry averaging. The models were validated using MOLPROBITY (89) implemented in the CCP4i2 interface (90).

### Molecular dynamics

Molecular dynamics was performed in YASARA v.20.12.24 (91). The structure of NagK complexed with GlcNAc and AMP-PNP was cleaned to remove water and PEG molecules. Molecular dynamics was run using the md_runfast macro for 5 ns using the AMBER15FB force field (92). Simulations including divalent cations were performed by replacing water molecule 97 with the relevant cation.

## Data availability

All data underpinning this work are publicly available. Structure coordinates and structure factor files are deposited with the Protein Data Bank (accession numbers: 7PA1, 7P7I, 7P7W, 7P9L, 7P9P and 7P9Y). Enzymatic and biophysical data are available as Supplementary Files or from Open Research Exeter (doi: to be confirmed on acceptance).

## Supporting information

This article contains supporting information (34, 42, 51, 68, 73, 93, 94, 95, 96, https://pymol.org/2/).

## Conflict of interest

The authors declare that they have no conflict of interest with the contents of the article.
