# Supplementary material for: The ROK kinase N-acetylglucosamine kinase uses a sequential random enzyme mechanism with successive conformational changes upon each substrate binding
Source: J Biol Chem. 2023 Feb 16;299(4):103033. doi: 10.1016/j.jbc.2023.103033 (PMC10031466; doi:10.1016/j.jbc.2023.103033)
Supplement: Supporting Table S1 and Figures S1–S12 [file mmc1.docx]

Roy et al.

Structure and function of *N*-acetylglucosamine kinase illuminates the catalytic mechanism of ROK kinases

Supplementary information

**Figure S1: Purification of NagK from diverse organisms**. Proteins were expressed in *E. coli* as 6-His-SUMO (*V. vulnificus* and *Plesiomonas shigelloides*) and 6-His-GST fusion proteins. Proteins were purified using immobilized metal affinity chromatography and size exclusion chromatography (SEC) using an ÄKTAxpress system. Samples from size exclusion were analyzed by SDS-PAGE to verify the identity and purity of the proteins. Samples were mixed 1:1 with loading buffer and run on ExpressPlus 4-20% gels (Genscript # M42015) using the manufacturer’s supplied MOPS buffer at 150 V for 50 minutes and stained with InstantBlue (Abcam #ab119211). Samples: M: 5 μL Spectra Multicolor Broad Range Protein Ladder (Thermo Scientific # 26623); C: 2 μL resuspended cells; P; 2 μL post-lysis pellet; S: 2 μL post-lysis supernatant; FT: 3 μL IMAC flow-through; SEC samples: 10 μL from samples at the SEC peak. **A**: 6-His-SUMO-NagK from *P. shigelloides*. **B**: 6_His-GST-NagK from *Photobacterium damselae*. **C**: 6-His-GST-NagK from *Pseudoalteromonas* sp. P1-8. **D**: 6-His-SUMO-NagK from *Vibrio vulnificus*.


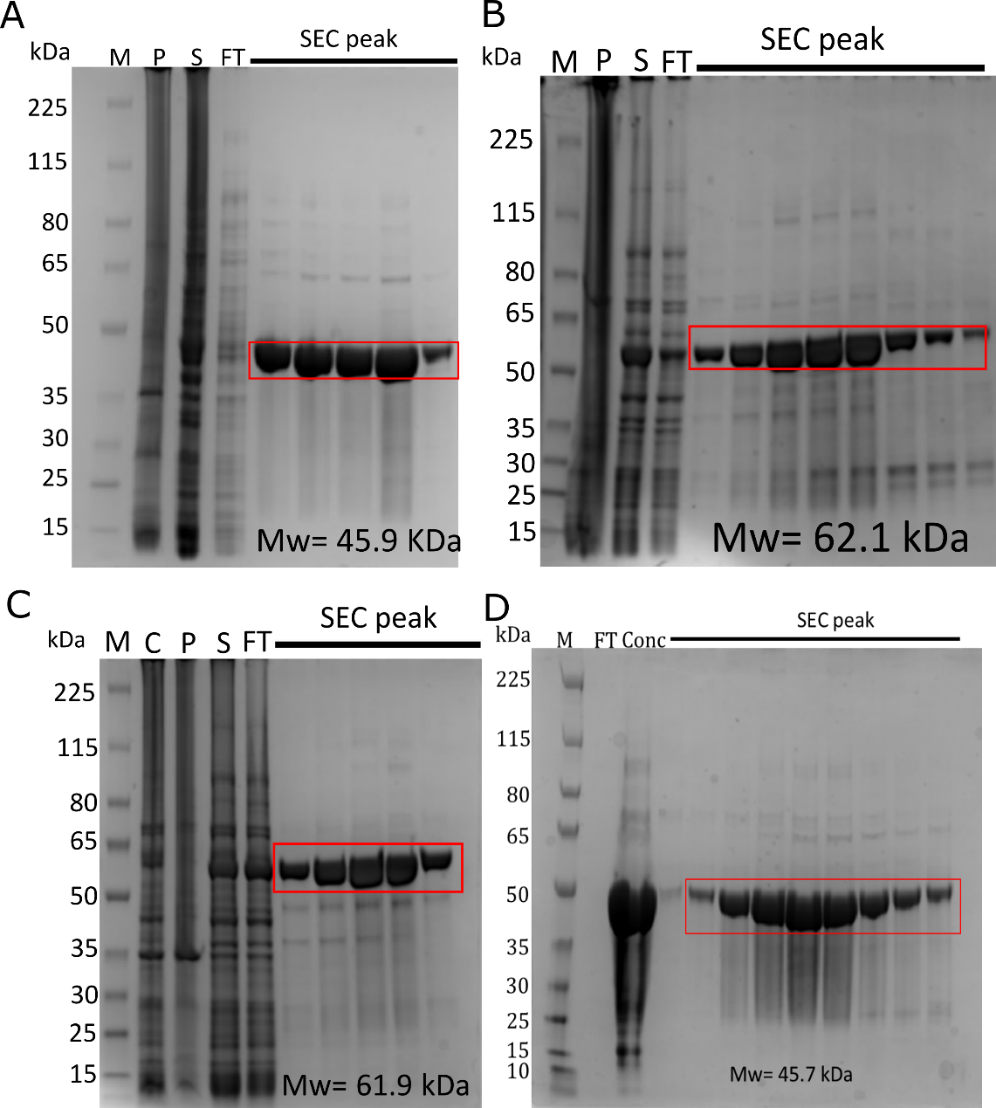


**Figure S2: Verification of NagK protein identity**. Purified NagK from the experiments above was separated by SDS-PAGE and western blot. The blot was probed using an iBind (Thermo Scientific) following the manufacturer’s instructions with mouse anti-penta-His (Qiagen # 34650) primary and IRDye® 680RD Goat anti-Mouse (LI-COR # 926-68070) secondary antibodies used at 1:2000 and 1:5000 dilutions respectively.


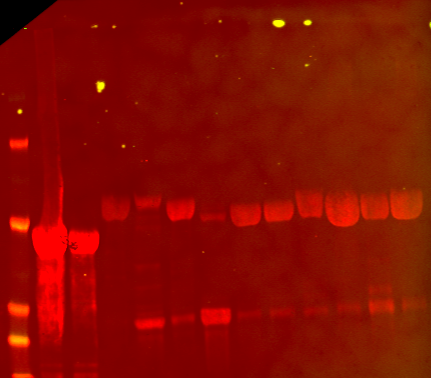


50

30

35

25

65

80

M

VvNAGK

PsNAGK

PdNAGK

YpNAGK

PaNAGK

62 kDa

46 kDa

**Figure S3: NagK products are weak inhibitors.** Product inhibition of NagK by GlcNAc-6-phosphate and ADP were assayed. Inhibition by GlcNAc-6-phosphate (**A**) was tested using the same assay as used for GlcNAc activity, using NagK at 180 ng/mL, GlcNAc at 100 μM and ATP at 300 μM; ADP inhibition was measured using a coupled assay with the enzymes *N*-acetylglucosamine-phosphate mutase (NagP; from *Candida albicans*; (1)), *N*-acetylglucosamine-1-phosphate uridyltransferase (GlmU; from *Escherichia coli*; (2)), and UDP-N-acetylgalactosamine dehydrogenase (WbpO; also has activity against UDP-GlcNAc; from *Pseudomonas aeruginosa* serotype O6; (3)) (**B**). This latter assay shows a reduced apparent rate, likely due to only partial turnover of substrates by the coupling enzyme WbpO. Each enzyme was purified in the same manner as described for NagK. The assay mixture contained 40 mM Hepes pH 7.5, 100 mM KCl, 8 mM MgCl_2_, 50 mM NH_4_(SO_4_)_2_, 5 mM DTT, 100 μg/mL BSA, 300 μM ATP, 100 μM GlcNAc, 200 μM UTP, 5 mM NAD^+^, 1 μg/mL NagK, 20 μg/mL NagP, 5 μg/mL GlmU, and 20 μg/mL WbpO. The reaction was followed at 340 nm for 2000 s and the rate determined following the lag phase. Data were fitted to the revised Morrison Ki equation (4) using GraphPad Prism v.9.4. The Morrison Ki for GlcNAc-6-phosphate was determined as 33 ± 4 mM, whilst the determined Ki for ADP was 4.7 ± 0.4 mM.


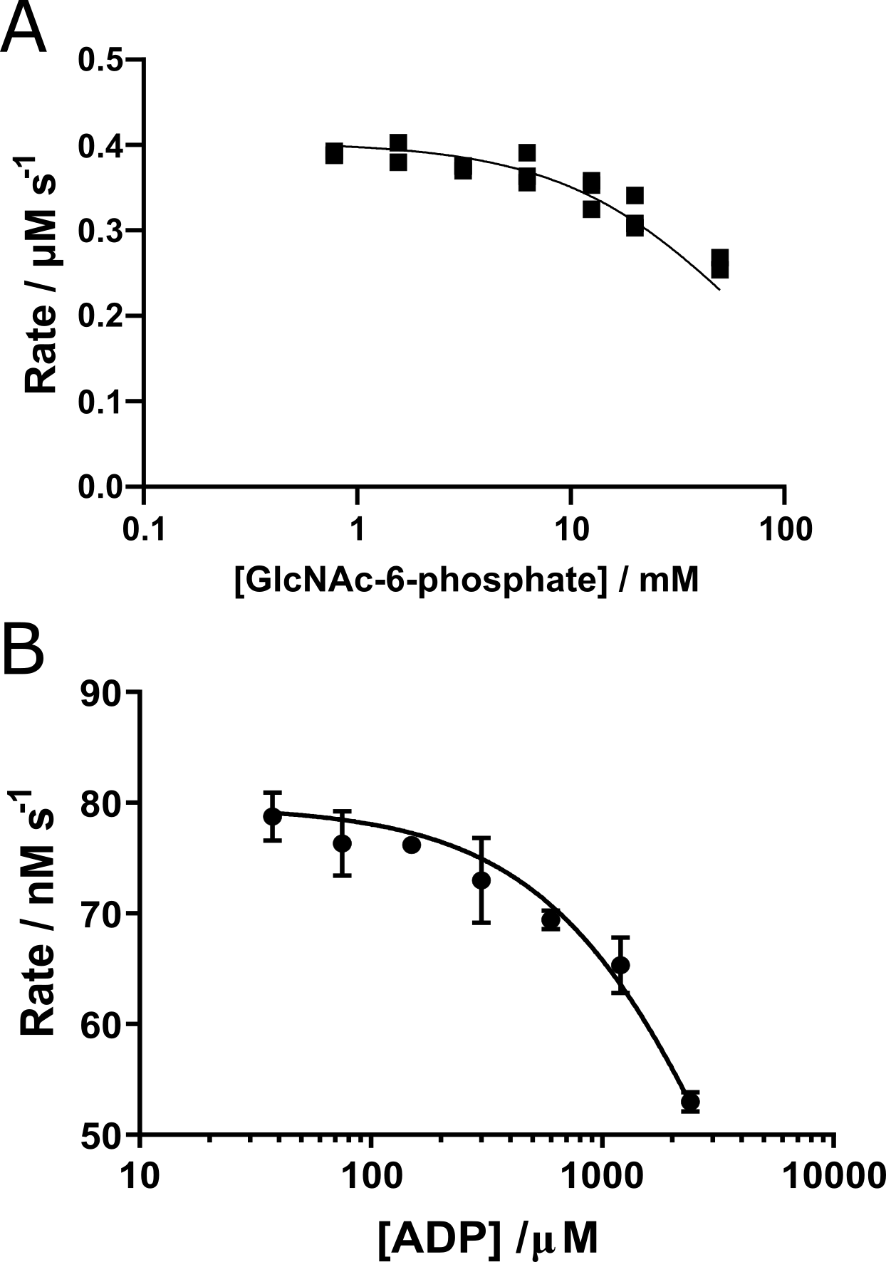


**Figure S4: NagK likely adopts a random equilibrium mechanism.** The mechanism of enzyme reactions can be determined by considering the effect of the reciprocal substrate on *K*’ (apparent *K_M_*) (5). The data from Figure 2C were analyzed to calculate apparent *K’* for both substrates at constant values of the partner substrate. For an ordered sequential mechanism, a decrease in *K’* would be expected as partner substrate concentration increases. Although the data are noisy, there is no evidence of such a decrease. The data therefore support a random equilibrium mechanism.

**Figure S5: Example data from differential scanning fluorimetry.** An example series is shown of NagK from *Plesiomonas shigelloides* with varying concentrations of GlcNAc. Protein was prepared at 0.1 mg/mL in 10 mM Hepes pH 7.5, with 8X SYPRO Orange. Data were collected in a QuantStudio6 qPCR machine and analyzed using Protein Thermal Shift software v1.4 (Applied Biosystems). **A:** Raw fluorescence data. **B:** Calculated derivative data. NagK displays a monotonic unfolding, with increasing concentrations of ligand increasing the melting temperature.

**
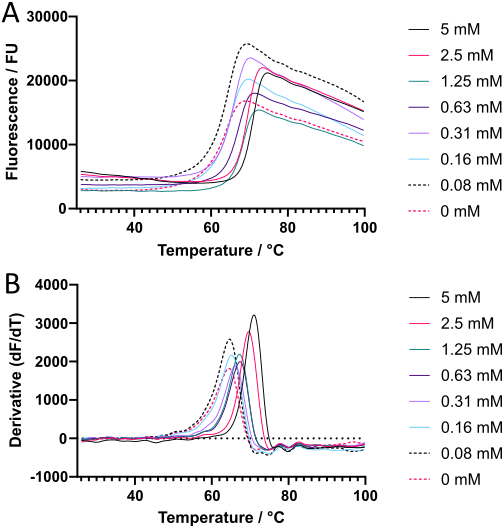
**

**Figure S6: NagK is active with all divalent cations that support coupling enzyme activity, with cobalt providing the best balance of low NagK background and coupling enzyme activity. Upper:** Activity data for the coupling enzymes pyruvate kinase and lactate dehydrogenase (0.2 U/mL), in a reaction with substrates 1 mM ADP, 200 µM NADH, and 500 µM phosphoenolpyruvate. The eight divalent cations that have previously supported enzyme activity were tested at 1-8 mM for activity. The activity is tracked by a reduction in absorbance at 340 nm (where NADH absorbs but the product NAD^+^ does not). A complete reaction results in a reduction of absorbance at 340 nm of ~0.7 AU. Several ions also absorb at 340 nm, resulting in higher backgrounds; iron, cobalt, and copper were tested at lower concentrations due to high absorbance. Magnesium and manganese support activity of the coupling enzymes at a fast rate, with the absorbance dropping to the background level (0.1 AU) either before the experiment could be measured or shortly afterwards. Nickel, iron, and cobalt support activity at a lower rate, showing a reduction in absorbance at 340 nm of 0.7-0.8 over the course of the experiment. Copper, zinc, and calcium show no change in absorbance. **Lower:** Activity data for NagK (5.1 nM) with the coupling enzymes pyruvate kinase and lactate dehydrogenase (1 U/mL), in a reaction with substrates 2 mM GlcNAc, 1 mM ATP, 200 µM NADH, and 500 µM phosphoenolpyruvate. Activity is tracked as in the upper panel. All five of magnesium, manganese, cobalt, nickel, and iron support NagK function as well as the coupling enzymes, showing a reduction in absorbance at 340 nm over the course of the experiment. Cobalt provides the lowest activity to NagK in the conditions tested: with cobalt the reaction fails to reach completion (reduction in absorbance of ~0.7 AU) over the course of the experiment. All data show the mean and standard error of three experimental replicates. Data are compiled from experiments conducted on different days and are representative of at least two experiments conducted on different days.

**Figure S7: Binding of ATP to generate a ternary complex requires a further rotation of the small domain, which is maintained in product complexes.** **A)** Structure of *apo*-*Ps*NagK (PDB ID: 7P7I), indicating the small domain in green (left) and the large domain in sky blue (right). **B)** Binding of GlcNAc (magenta, GlcNAc carbon atoms colored yellow; PDB ID: 7P9Y) induces a large rotation of the small domain relative to the large domain (image identical to Fig 4B). **C)** Upon binding of AMP-PNP (dark orange; AMP-PNP shown as spheres, carbon atoms colored white, PDB ID: 7P9P), the small domain (left) rotates relative to the large domain. There is a 16° rotation from the position of the GlcNAc complex to the GlcNAc-AMP-PNP complex. **D)** Binding of AMP-PNP alone (light orange, PDB ID: 7PA1) to NagK does not cause a significant rotation of the small domain. **E, F)** Product complexes with either the abortive complex of NagK with GlcNAc and ADP (**E**, yellow, PDB ID: 7P7W) or the complex of NagK with GlcNAc-6P (**F**, marine blue, PDB ID: 7P9L) are very similar in conformation to the ternary complex of NagK with GlcNAc and AMP-PMP (dark orange). Structures were superimposed over the large domain (amino acids 105-290). Atom colors: nitrogen, blue; oxygen, yellow; phosphorus, orange; chloride, green; potassium, purple. Figure generated using PyMOL v. 2.4.1 (6).


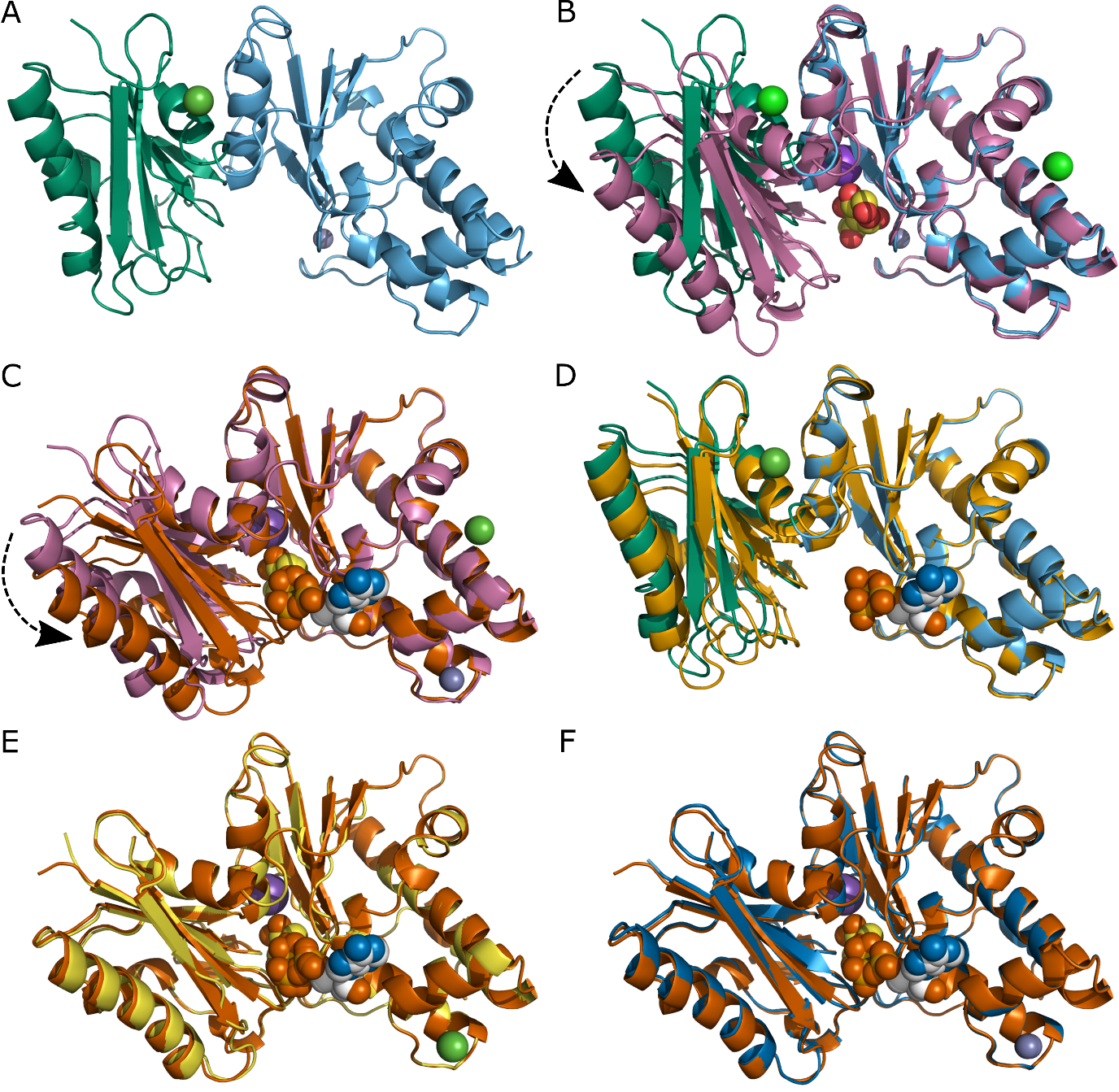


**Figure S8: Sequence alignment of NagK orthologues.** The sequences chosen share 39-78% sequence identity. Possible active site residues are indicated: dark blue arrow, proposed metal binding site (D6); orange box, ATP phosphate binding region (G9-K11); orange arrows, side chain ATP binding (T132, E196); sky blue arrow, main chain ATP binding (G255); green arrows, GlcNAc binding (S78, N104, E154, H157, D187); black arrow, proposed catalytic base (D105). Figure produced using ESpript v.3.0 (7).**
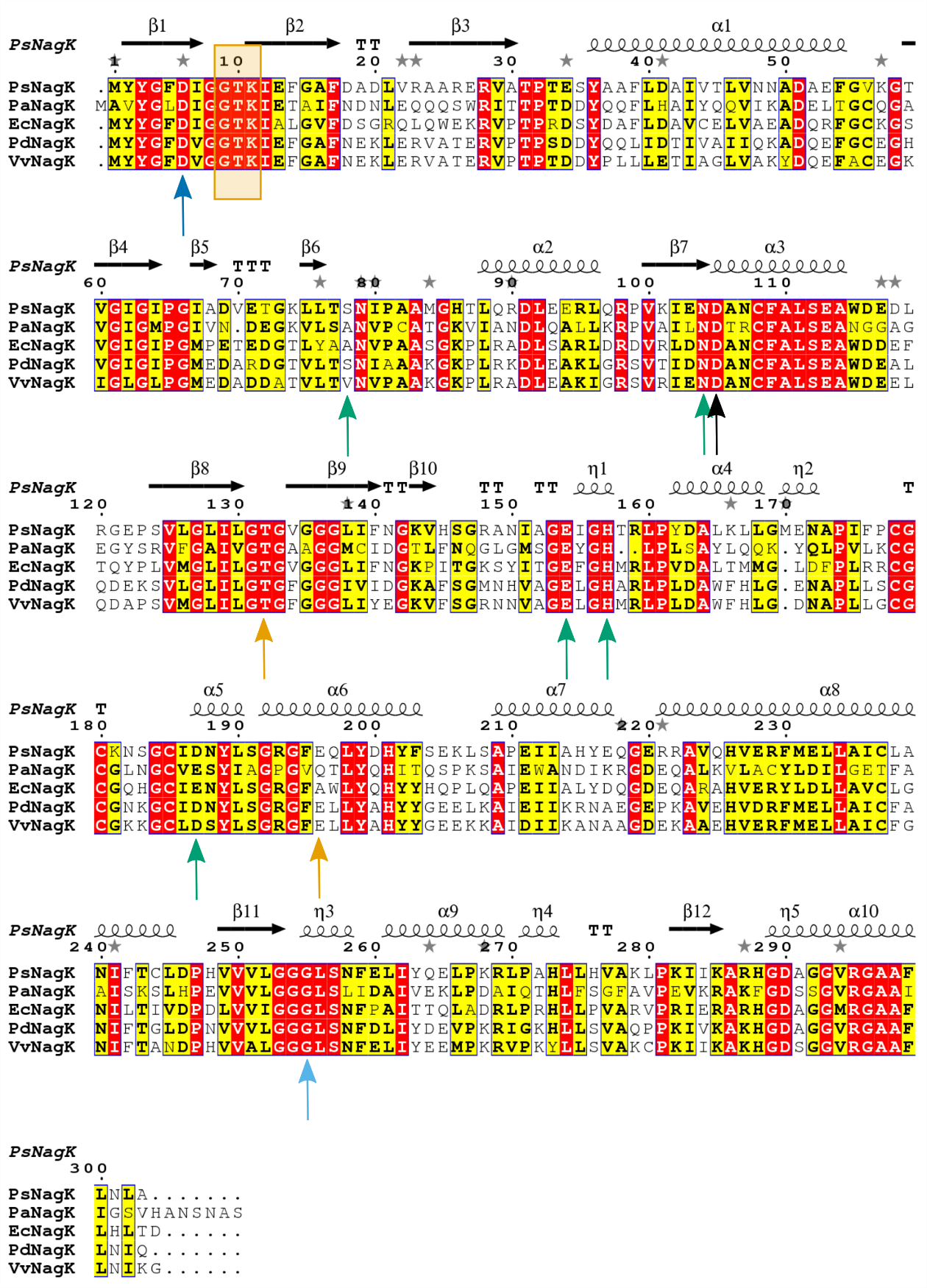
**

**Figure S9: The NagK ternary complex with GlcNAc and AMP-PNP shows the same conformation as a previous ROK kinase ternary complex including the catalytic metal.** The structure of NagK in complex with AMP-PNP (black dashed arrow) and GlcNAc (black solid arrow) (**A**; PDB ID: 7P9P) was superimposed with the structure of human NanK in complex with ADP (grey dashed arrow), ManNAc (grey solid arrow) and magnesium (black arrowhead) (**C**; PDB ID: 2YHY) using Pymol v2.5, focusing on the larger domain (right in this image). The superposition (**B**) shows that the conformation of the small domain relative to the large domain is well conserved between the two structures. The magnesium position is consistent with this being the catalytic cation position in the NagK structure. Colors: *Ps*NagK: green; *Hs*NanK: sky blue; nitrogen atoms: blue; oxygen atoms: red; phosphorus atoms: orange spheres; zinc ions: grey spheres; magnesium/calcium ions: light green spheres; potassium ions: purple spheres. Images generated using Pymol v 2.5 (6).

**
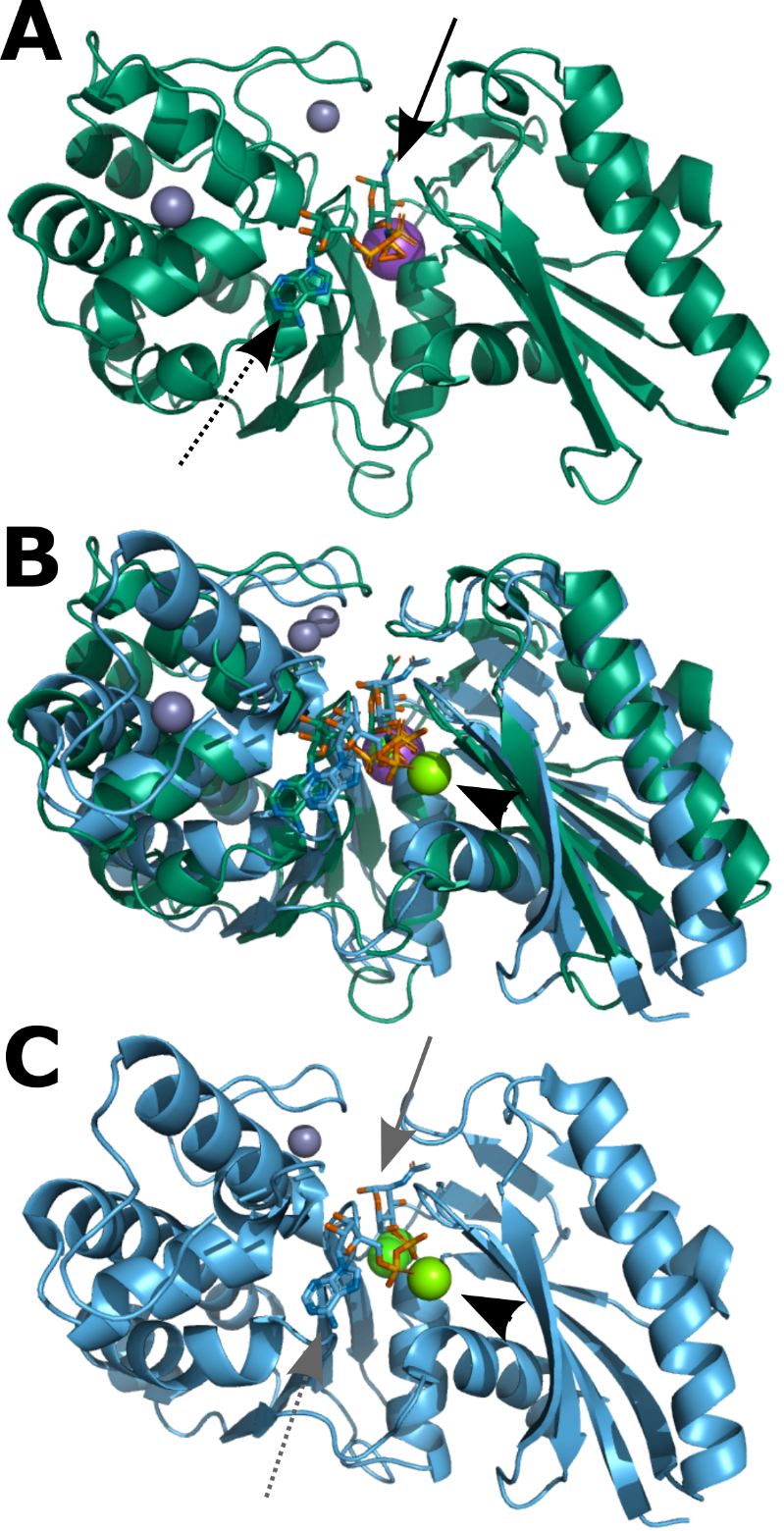
**

**Figure S10: Molecular dynamics confirms the likely metal binding site.** The NagK-GlcNAc-AMP-PNP ternary complex (PDB ID: 7P9P) was altered to replace the nitrogen in AMP-PNP with oxygen (A). In separate structures, a bound water between D6 and AMP-PNP was replaced with magnesium (B), calcium (C) or manganese (D). Molecular dynamics was run for 5 ns using YASARA v.20.12.24. Images show the proposed metal binding site, with amino acids within 4.5 Å of the metal shown as sticks and the remainder of NagK as cartoon. Figure generated using PyMOL v. 2.4.1 (6).

**
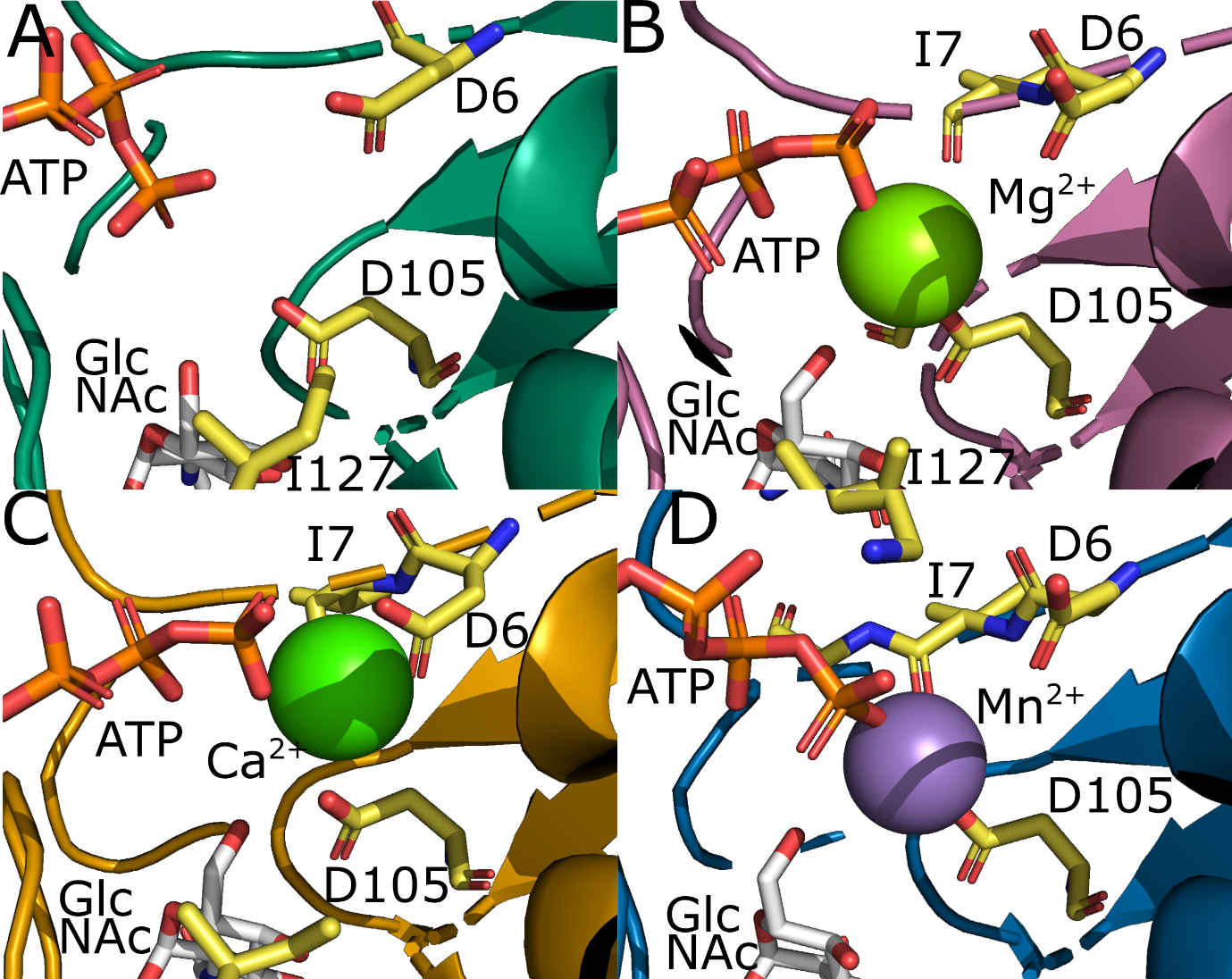
**

**Figure S11: Sequence alignment of ROK kinases.** Sequences of six ROK kinases representing the major known activities were aligned, using available crystal structures. 3OHR: fructokinase from *Bacillus subtilis* (8); 2AA4: *N*-acetylmannosamine kinase from *E. coli*; 3HTV: d-allose kinase from *E. coli*. 3VGL: glucokinase from *Streptomyces griseus* (9); 1WOQ: polyphosphate glucokinase from *Arthrobacter* sp. KM (10). NagK proposed active site residues are indicated: dark blue arrow, proposed metal binding site (D6); orange box, ATP phosphate binding region (G9-K11); orange arrows, side chain ATP binding (T132, E196); sky blue arrow, main chain ATP binding (G255); green arrows, GlcNAc binding (S78, N104, E154, H157, D187); black arrow, proposed catalytic base (D105). Figure produced using ESpript v.3.0 (7).

**
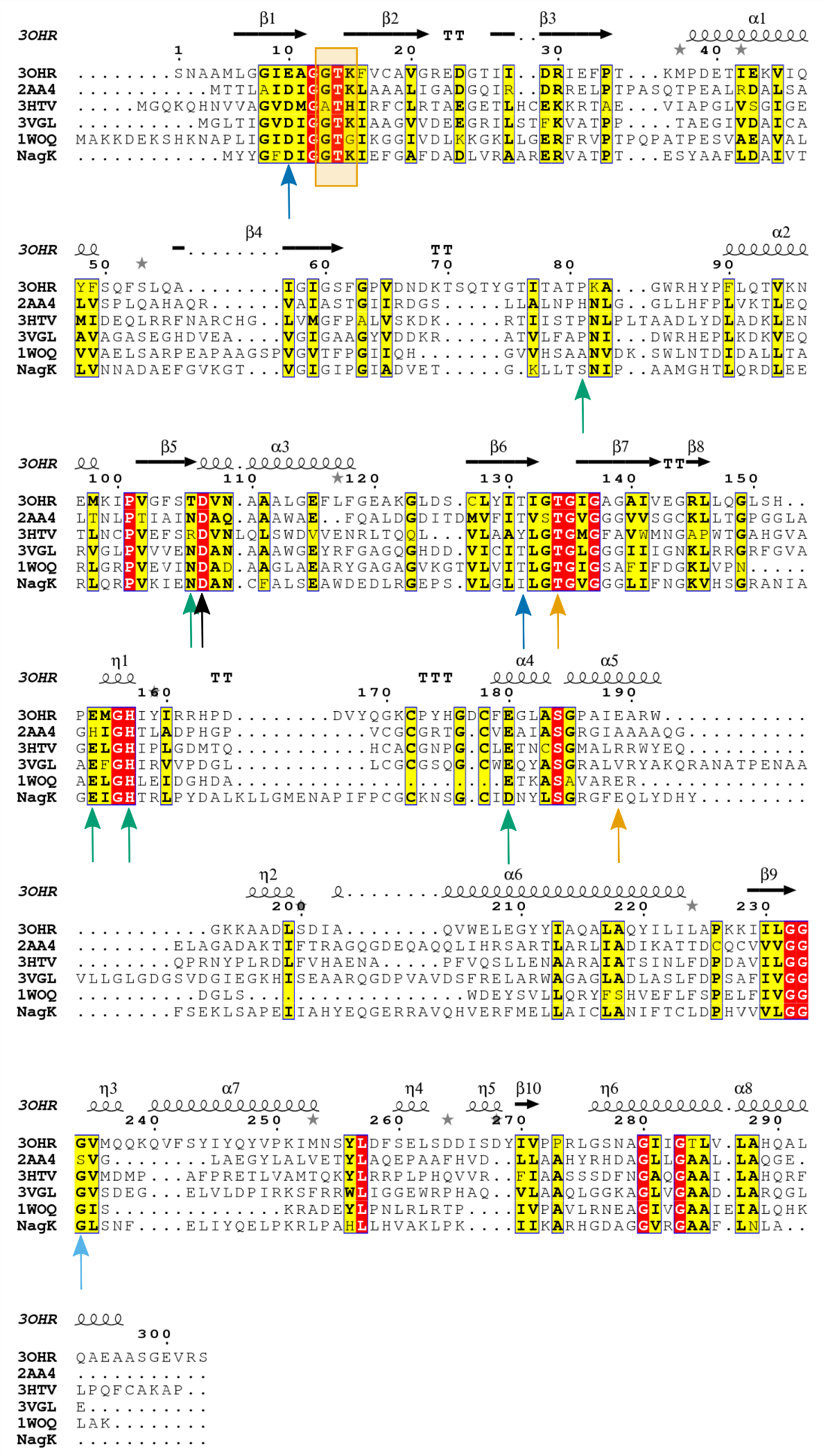
**

**Figure S12: Differential scanning fluorimetry suggests that the NagK mutants tested do not significantly affect protein structure.** Wild-type NagK and the eleven mutants experimentally tested for NagK activity were unfolded using differential scanning fluorimetry. The melting temperatures were determined using the Boltzmann method using Protein Thermal Shift Software v. 1.4 (Applied Biosystems). Ten experimental replicates were taken for each mutant. The wild-type (T_m_ -= 73.4 °C) and all mutants show a T_m_ of over 70 °C except for the N104D and N104D/E156Q double mutan, which both showed a reduction in Tm to 65-67 °C. This likely reflects the addition of a second acid group directly adjacent to D105 and loss of a hydrogen bond between N104 side chain and a main chain carbonyl. DSF for all mutants gave a very clear and reproducible unfolding pattern, suggesting that the proteins are well folded. Image generated with Graphpad v. 9.5.0.

| NagK/NagK-substrate complex | Morpheus screen position/conditions | Co-crystallized | Soaking substrate | Soaking/cryoprotection solution | Soaking time (sec) |
| --- | --- | --- | --- | --- | --- |
| NagK (Native) | A12  (60 mM divalent cations; 0.1 M Tris/bicine pH 8.5; 12.5% each MPD, PEG 1K, PEG 3350 | N/A | N/A | 25% (v/v) MPD, 20% (w/w) PEG 1K, 20% (w/w) PEG 3350, 50 mM Tris pH 8.5 | N/A |
| NagK with GlcNAc | B8  (90 mM halogens; 0.1 M Na-HEPES/MOPS pH 7.5; 12.5% each MPD, PEG 1K, PEG 3350) | 500 µM GlcNAc | N/A | 25% (v/v) MPD, 20% (w/w) PEG 1K, 20% (w/w) PEG 3350, 50 mM MOPS pH 7.5 | N/A |
| NagK with GlcNAc and AMP | D7  (120 mM alcohols; 0.1 M Na-HEPES/MOPS pH 7.5; 30% each glycerol and PEG 4K) | 500 µM GlcNAc | 10 mM AMP | 30% (v/v) glycerol, 15% (w/w) PEG 4K, 100 mM MOPS pH 7.5 | 30 |
| NagK with GlcNAc and AMP-PNP | A3  (60 mM divalent cations; 0.1 M imidazole/MES pH 6.5; 30% each glycerol and PEG 4K) | 500 µM GlcNAc | 10 mM AMP-PNP | 30% (v/v) glycerol, 15% (w/w) PEG 4K, 100 mM imidazole pH 6.5 | 90 |
| NagK with GlcNAc and ADP | D3  (120 mM alcohols; 0.1 M imidazole/ MES pH 6.5; 30% each glycerol and PEG 4K) | 500 µM GlcNAc | 10 mM ADP | 30% (v/v) glycerol, 15% (w/w) PEG 4K, 100 mM imidazole pH 6.5 | 60 |
| NagK with GlcNAc-6-phosphate | B9  (90 mM halogens; 0.1 M Tris/bicine pH 8.5; 30% each PEG 550 MME and PEG 20K) | 10 mM GlcNAc-6-phosphate and 500 µM ATP | N/A | 35% PEG 500-MME, 15% PEG 8000, 100 mM Tris pH 8.5, 10 mM ATP | 60 |
| NagK with AMP-PNP | C9  (0.09M NPS; 0.1 M Tris/bicine pH 8.5; 30% each PEG 550 MME and PEG 20K) | N/A | 10 mM AMP-PNP | 30% (v/v) PEG400, 15% (w/w) PEG 4K, 100 mM Tris pH 8.5 | 90 |

**Table S1: Crystallization conditions**

**References:**

1. Nishitani, Y., Maruyama, D., Nonaka, T., Kita, A., Fukami, T. A., Mio, T., Yamada-Okabe, H., Yamada-Okabe, T., and Miki, K. (2006) Crystal structures of N-acetylglucosamine-phosphate mutase, a member of the alpha-D-phosphohexomutase superfamily, and its substrate and product complexes. *J Biol Chem* **281**, 19740-19747

2. Olsen, L. R., Vetting, M. W., and Roderick, S. L. (2007) Structure of the E. coli bifunctional GlmU acetyltransferase active site with substrates and products. *Protein Sci* **16**, 1230-1235

3. Zhao, X., Creuzenet, C., Belanger, M., Egbosimba, E., Li, J., and Lam, J. S. (2000) WbpO, a UDP-N-acetyl-D-galactosamine dehydrogenase from Pseudomonas aeruginosa serotype O6. *J Biol Chem* **275**, 33252-33259

4. Cook, P. F., and Cleland, W. W. (2007) *Enzyme kinetics and mechanism*, Garland Science, London ; New York

5. Marangoni, A. G. (2002) Two-Substrate Reactions. in *Enzyme Kinetics*. pp 90-101

6. The PyMOL Molecular Graphics System. 2.4.1 Ed., Schrödinger, LLC.

7. Robert, X., and Gouet, P. (2014) Deciphering key features in protein structures with the new ENDscript server. *Nucleic Acids Res* **42**, W320-324

8. Nocek, B., Stein, A. J., Jedrzejczak, R., Cuff, M. E., Li, H., Volkart, L., and Joachimiak, A. (2011) Structural studies of ROK fructokinase YdhR from Bacillus subtilis: insights into substrate binding and fructose specificity. *J Mol Biol* **406**, 325-342

9. Miyazono, K., Tabei, N., Morita, S., Ohnishi, Y., Horinouchi, S., and Tanokura, M. (2012) Substrate recognition mechanism and substrate-dependent conformational changes of an ROK family glucokinase from *Streptomyces griseus*. *J Bacteriol* **194**, 607-616

10. Mukai, T., Kawai, S., Mori, S., Mikami, B., and Murata, K. (2004) Crystal structure of bacterial inorganic polyphosphate/ATP-glucomannokinase. Insights into kinase evolution. *J Biol Chem* **279**, 50591-50600
